# Projections of the Diencephalospinal Dopaminergic System to Peripheral Sense Organs in Larval Zebrafish (*Danio rerio*)

**DOI:** 10.3389/fnana.2018.00020

**Published:** 2018-03-19

**Authors:** Melanie Haehnel-Taguchi, António M. Fernandes, Margit Böhler, Ina Schmitt, Lena Tittel, Wolfgang Driever

**Affiliations:** ^1^Developmental Biology, Faculty of Biology, Institute Biology I, Albert-Ludwigs-University Freiburg, Freiburg, Germany; ^2^Department Genes-Circuits-Behavior, Max Planck Institute of Neurobiology, Martinsried, Germany; ^3^BIOSS-Centre for Biological Signaling Studies, Freiburg, Germany

**Keywords:** dopamine, zebrafish, diencephalospinal dopamine system, A11 dopaminergic group, lateral line organ, lateral line ganglion, inner ear, trigeminal ganglion

## Abstract

Dopaminergic neurons of the descending diencephalospinal system are located in the posterior tuberculum (PT) in zebrafish (*Danio rerio*), and correspond in mammals to the A11 group in hypothalamus and thalamus. In the larval zebrafish, they are likely the only source of central dopaminergic projections to the periphery. Here, we characterized posterior tubercular dopaminergic fibers projecting to peripheral sense organs, with a focus on the lateral line neuromasts. We labeled and identified catecholaminergic neurons and their projections by combining two immunofluorescence techniques, (i) using an antibody against Tyrosine hydroxylase, and (ii) using an antibody against GFP in transgenic zebrafish expressing in catecholaminergic neurons either membrane-anchored GFP to track fibers, or a Synaptophysin-GFP fusion to visualize putative synapses. We applied the CLARITY method to 6 days old whole zebrafish larvae to stain and analyze dopaminergic projections by confocal microscopy. We found that all lateral line neuromasts receive direct innervation by posterior tubercular dopaminergic neurons, and tracked these projections in detail. In addition, we found dopaminergic fibers projecting to the anterior and posterior lateral line ganglia, and extensive central dopaminergic arborizations around the terminal projection field of the lateral line afferent neurons in the hindbrain medial octavolateralis nucleus (MON). Therefore, dopaminergic innervation may affect lateral line sense information at different processing stages. Additional dopaminergic fibers innervate the trigeminal ganglion, and we observed fine catecholaminergic fibers in the skin with arborization patterns similar to free sensory nerve endings. We also detected potentially dopaminergic fibers innervating inner ear sensory epithelia. Therefore, the diencephalospinal A11-type dopaminergic system may broadly modulate peripheral senses. We also briefly report peripheral sympathetic catecholaminergic projections labeled in our experiments, and their innervation of the developing intestine, swim bladder and abdominal organs.

## Introduction

Recent studies have revealed that dopaminergic (DA) neurons in the posterior tuberculum (PT) and ventral diencephalon of larval zebrafish correspond to the mammalian dopaminergic A11 system, which all depend on the transcription factor Orthopedia (Otp) (Ryu et al., 2007; Fernandes et al., 2013). The mammalian A11 system is located in the dorsal hypothalamus and caudal thalamus (Smeets and González, 2000) and is the only group of DA neurons sending projections into the spinal cord (Fleetwood-Walker et al., 1988), and plays a role in the neurological disorder Restless-Legs-Syndrome (RLS), in which patients experience unpleasant sensations in the legs (Clemens et al., 2006). The diencephalic DA systems in zebrafish comprise the A11 homologous groups (PTar, PTac, PTp, PTN; previously named DC2, DC4-6), as well as groups in the preoptic region, the pretectum, the ventral thalamus, and several hypothalamic groups (Rink and Wullimann, 2001). The catecholaminergic projections in the larval zebrafish central nervous system have been previously characterized (Rink and Wullimann, 2002; McLean and Fetcho, 2004; Tay et al., 2011), and the A11 homolog groups (PTar, PTac, PTp, PTN) have been shown to extend fibers to the subpallium, endohypothalamic tract (eht), rhombencephalon (RhE) and spinal cord (Table 1; Kastenhuber et al., 2010; Tay et al., 2011). There is also evidence that the descending DA system of larval zebrafish projects to peripheral sense organs. Particularly, neurons with large cell bodies in the anterior PT appear to contact the neuromasts of the lateral line (Bricaud et al., 2001; Jay et al., 2015; Toro et al., 2015). However, there is to date no detailed anatomical analysis of DA fibers projecting into the periphery.

**Table 1:** Ventral diencephalic dopaminergic clusters (*Introduction*).

**Name**	**Description**	**Group**	**Homology**	**Mol. marker**	**Cells and anatomical position**	**Known projections**
PTar	Anterior PT rostral group	DC2	A11	*otp*	Large, pear-shaped, in peri-ventricular nucleus of PT	Endohypothalamic, ascending: SP descending: Rh, SC
PTac	Anterior PT caudal group	DC4	A11	*otp*	Large, in periventricular nucleus of caudal PT	Endohypothalamic, descending: Rh, SC
PTp	Posterior PT posterior group	DC5	A11	*otp*	Small round cells of peri-ventricular nucleus of PT	Fewer or no connections to the SC
PTN	Posterior tuberal nucleus	DC6	A11	*otp*	Small round cells of posterior tuberal nucleus	Few or no connections to the SC
Hdm	Dorsal medial hypothalamus	DC3	A14	*nkx2.1*	Small round liquor contacting cells	Local, hypothalamic
				*nkx2.2*		
Hc	Caudal hypothalamus	DC7	A14	*nkx2.1*	Small cells in caudal hypothalamus	Local, hypothalamic

*Groups of DA neurons in the diencephalon named according to their anatomical location. Molecular markers suggest homology to mammalian DA groups (Ryu et al., 2007; Yamamoto and Vernier, 2011). Zebrafish DA clusters (DC) have previously been numbered from rostral to caudal (Rink and Wullimann, 2001). Far projecting neurons send descending projections into the rhombencephalon, spinal cord and to peripheral sensory organs. nkx 2.1, NK2 homeobox 1 transcription factor; otp, Orthopedia transcription factor; PT, posterior tuberculum; Rh, rhombencephalon; SC, spinal cord; SP, subpallium*.

Studies of the diencephalospinal DA system in zebrafish point to functions in sensory and motor system modulation. DA modulation has been proposed in sensory-motor gating (Burgess and Granato, 2007), the selection of motor programs during development (Thirumalai and Cline, 2008; Lambert et al., 2012), in the visual modulation of audiomotor processing (Mu et al., 2012) and behavior selection (Yao et al., 2016). Further, it has been shown that the activity of the posterior tubercular groups of the diencephalic DA system is tuned to sensory stimuli, and that the lateral line system contributes to this sensory tuning (Reinig et al., 2017). Studies of the zebrafish lateral line system have already described efferent innervation of the posterior neuromasts by cell bodies residing in the diencephalon (Metcalfe et al., 1985), which appeared to be catecholaminergic (Bricaud et al., 2001). Recently, it was shown that dopamine release close to the hair cell ribbon synapse in lateral line neuromasts has an excitatory effect on hair cell transmission (Toro et al., 2015), while another study showed activity in posterior tubercular DA neurons projecting to the lateral line neuromasts correlated to motor activity during fictive swimming (Jay et al., 2015). Although, these findings point to a role of the posterior tubercular DA system in sensory-motor modulation, the exact function of DA modulation of sensory and motor targets is not fully understood.

Here, we investigated in detail peripheral catecholaminergic projections in larval zebrafish. We adapted the recently developed CLARITY method for tissue clearing (Chung and Deisseroth, 2013; Tomer et al., 2014; Treweek et al., 2015) for use in 6 day old zebrafish larval whole mounts to resolve the peripheral projection patterns of the larval central DA system. We focused on the lateral line but also considered connections to the inner ear and trigeminal system. Our data revealed extensive peripheral diencephalic DA projections to all larval anterior and posterior lateral line neuromasts. DA modulation of sensory information may not only occur at the sense organ, but also at the levels of the lateral line ganglia, and of the afferent lateral line projection fields into the medial octavolateralis nucleus (MON) in the rhombencephalon. DA neurons therefore are likely to play an important and diverse role in efferent modulation of sensory systems.

## Materials and methods

### Experimental animals and transgenic lines

Zebrafish (*Danio rerio*) were bred and maintained in our animal facility under standard conditions (Westerfield, 2000). Fish of the transgenic lines *Tg(th:Gal4-VP16)*^*m*1233^ and *Tg(UAS:eGFP-CAAX)*^*m*1230^ (Fernandes et al., 2012) were crossed to characterize the peripheral targets of DA neurons by membrane-tagged GFP. To visualize synaptic structures these fish were crossed with *Tg(UAS:SypGFP, clmc2:EGFP)*^*m*1238^. For this line the 5xUAS:Synaptophysin-EGFP encoding cassette (Meyer and Smith, 2006) was cloned into pDestTol2CG (Kwan et al., 2007), and a transgenic zebrafish line was generated using the Tol2 transposon system. Eggs were collected and treated with 0.2 mM phenylthiourea (PTU, Sigma, St. Louis, MO) to suppress pigmentation. Larvae were screened for GFP expression on days 3–4 using a stereomicroscope (Leica MZ 16F, Leica, Wetzlar, Germany). Embryos and larvae were raised at 28.5°C in Eggwater (0.3 g sea salt/L reverse osmosis water) until the age of 6 days post fertilization (dpf) when larvae were deep-anesthetized in 0.02% Tricaine (ethyl 3-aminobenzoate methanesulfonate; Sigma, St. Louis, MO) and fixed in hydrogel solution (CLARITY method). All experimental procedures were in accord with the German laws for animal care, and a permit was obtained from the Regierungspraesidium Freiburg.

### Immunofluorescence and antibody characterization

All washing and incubation steps were done on a shaker (Rotamax 120, Heidolph, Schwabach, Germany) or rotator (Roto-Torque 7637-0, Cole-Parmer, Vernon Hills, IL). All antibodies (Table 2) were previously characterized and pre-absorbed against wildtype embryos at stages without antigen expression to avoid unspecific binding.

**Table 2:** Antibodies (Materials And Methods).

**Antibody**	**Immunogen**	**Source (Cat. No.), species**	**RRID**	**Dilution**
**PRIMARY**				
GFP	Green Fluorescent Protein	Life technologies (A10262), chicken (monoclonal)	AB2534023	1:400
TH	Tyrosine Hydroxylase from zebrafish th1 gene fragment	(Ryu et al., 2007), rabbit (polyclonal)	AB2631248	1:500
Islet1/2	Recombinant fusion protein containing aa 178–349 of Isl1 protein (C-terminus)	DSHB (39.4D5), mouse (monoclonal)	AB2314683	2 μg/ml
**SECONDARY**				
Alexa 488	Chicken IgG	Life technologies (A11039), Goat	AB2534096	1:1,000
Alexa 555	Rabbit IgG	Life technologies (A21430), Goat	AB2535851	1:1,000
Alexa 555	Mouse IgG	Life technologies (A21425), Goat	AB2535846	1:1,000
Alexa 633	Rabbit IgG	Life technologies (A21070), Goat	AB2535731	1:1,000

### “CLARITY” method (adapted for 6 day-old zebrafish larvae)

CLARITY is a method to achieve transparency of intact tissue while preserving ultrastructure and fluorescence, allowing access of antibodies to native biological content by transforming biological tissue into a hydrogel-tissue hybrid (Chung et al., 2013; Tomer et al., 2014). We modified the protocol for the passive CLARITY technique, which can be used to clear whole organs or organisms (Treweek et al., 2015), to apply it to whole intact zebrafish larvae. In this study larvae were sacrificed and transferred to hydrogel solution at 6 dpf, however the protocol is suitable for 3–8 dpf larvae.

We prepared the hydrogel monomer (HM) stock solution (Table 3) on ice and distributed it to 15 ml falcon tubes to be stored at −20°C until needed. All ingredients and the solution were kept sufficiently cold during preparation. We also prepared the clearing solution (Table 3), which was stored at room temperature.

**Table 3:** Solutions used for passive CLARITY (Materials and Methods).

**Solution**	**Chemical**	**Supplier**	**Amount added**	**Final content**
Hydrogel	Acrylamide (40%)	Roth, Karlsruhe	10 ml	4%
Monomer	VA-044 Initiator	Wako, Osaka	0.25 g	0.25%
Solution (100 ml)	10x PBS	Lab made	10 ml	1x
	PFA (16%)	AppliChem, Darmstadt	25 ml	4%
	DI Water	Lab supply	55 ml	
Clearing	Boric Acid	AppliChem, St. Louis	12.4 g	200 mM
Solution (1 L)	SDS	Roth, Karlsruhe	40 g	4%
	DI Water		Fill to 1 L	
	NaOH		Adjust to pH 8.5	

For hydrogel embedding, a tube of HM solution was thawed on ice and added to a tube containing larvae treated with an overdose of Tricaine (0.02%), which was carefully removed before adding the HM solution to avoid diluting it. The sample was kept on ice until moved to a 4°C room overnight to allow diffusion of the HM solution into the tissue. To remove air (oxygen) from the tube containing the sample, the bottom of the desiccation chamber (vacuum desiccator “Space Saver,” Bel-Art Products, Wayne, NJ) was filled with dry ice and the whole chamber was placed in a container with warm water. The tube was placed on a rack on top of the ice to avoid freezing of the contents and the lid of the tube was twisted open to allow gas exchange. The vacuum pump was turned on for 10 min and then turned off, until the lid of the chamber opened and the ice was melted, leaving the chamber filled with CO_2_. The lid of the tube was immediately closed to avoid exposure to air. The tube was placed on a shaker for 2 h at 37°C to allow the acrylamide to polymerize with the biomolecules within the tissue. For tissue clearing larvae were removed from the hydrogel under a fume hood using a fine tool (e.g., hair of a brush attached to holder) to avoid tissue damage, transferred to a 50 ml tube with clearing solution (Table 3) and placed on a shaker for 5–7 days at 37°C, changing the clearing solution every other day. Before immunostaining, larvae were washed in PBST for 1–2 days. For immunostaining with CLARITY, specimens were blocked in blocking solution (PBST with 1% DMSO [PBSTD], 2% BSA and 5% goat serum) for 2–3 h at room temperature before the primary antibodies (Table 2) in blocking solution were added for 2–4 days at 4°C. Larvae were washed 5–6 times in PBSTD before secondary antibodies (Table 2) in PBSTD were added for 2–3 days at 4°C. If no TOTO-3 labeling was performed, larvae were washed 1–2 times in PBST and then stored in 80% glycerol until imaging. For TOTO-3 labeling larvae were washed in PBST 4 times for 15 min and incubated with TOTO-3 (TOTO-3-Iodite, Molecular Probes Invitrogen T3604, Eugene, OR, 1:2,000 in PBST) overnight at room temperature and then washed 3 times in PBST before transferring to glycerol. Specimens could be stored in glycerol at 4°C at least for 2 weeks without quality loss.

### Vital dye labeling of the lateral line system

Hair cells in neuromasts of the lateral line system were labeled by incubating live larvae in 5 μM 2-[4(Dimethylamino)styryl]-1-ethylpyridinium iodide (DASPEI, Sigma-Aldrich, St. Louis, MO) in Eggwater for 20 min. Larvae were washed 3 times in Eggwater and mounted in 1.2% low melting agarose (ThermoFisher, Waltham, MA) on the lid of a small Petri dish for visual inspection of the lateral line system and further dye injections.

### Anterograde/retrograde labeling

To achieve tracing of lateral line afferent and efferent neurons, all cells of a neuromast, including primary afferent and efferent neurons, were labeled by dye-bolus injections in a similar way as previously described (Alexandre and Ghysen, 1999) with some modifications. Briefly, 5–6 dpf larvae labeled with DASPEI, as described above, and anesthetized with 0.16 mg/ml Tricaine in Eggwater were mounted in 1.2% low melting agarose. They were placed under an epifluorescence microscope (Leica MZ 16F, Leica, Wetzlar, Germany) and the DASPEI-labeled neuromasts were observed using an RFP filter. A few microliters of water were added to crystals of Rhodamine dextran (Dextran, Tetramethylrhodamine, biotinylated, 10,000 MW, lysine fixable, “mini-ruby,” ThermoFisher, Waltham, MA), such that the crystals dissolved in the water and the paste was allowed to dry on a glass slide. The dried paste was stored in the freezer. By adding 0.5 μl of water to the dried paste, minute amounts could be picked up with the tip of a glass micro-pipette to form a dye-bolus (tip was broken to ~10 μm diameter). Branches of the lateral line nerves were labeled by making a small lesion with the dye-loaded pipette at the location of a neuromast. Larvae were transferred to dishes with Eggwater and left at least 6 h at 28.5°C or overnight, until the dye had filled the entire neurons.

### Microscopy and image processing

Overview images of the DASPEI-labeled lateral line system were taken with an epifluorescence microscope (Axio Examiner, Zeiss, Oberkochen, Germany) using a DSRed filter. For confocal microscopy larvae were mounted in mounting medium (Vectashield, Vector Laboratories, Burlingame, CA; and 1% agarose) on custom built microscopy slides made from a 24 × 60 mm coverslip and separated by three layers of 18 × 18 mm coverslips (Menzel-Glaeser, Braunschweig, Germany) from the top coverslip of 0.17 mm thickness (Hecht-Assistent, Sondheim, Germany), sealed with nail polish and stored in the dark at 4°C until imaging. Larvae were observed using an upright Zeiss LSM-510 confocal microscope equipped with a 25x/0.8NA objective (LD LCI Plan-Apochromat 25x/0.8 DIC, immersion corrected) with glycerol immersion and 488, 561, and 633 nm lasers, using the Zeiss ZEN Black acquisition software. Z-stacks were scanned with 1 μm step size and 1,024 × 1,024 or 512 × 512 pixels per image. Tiles were scanned with 10% image overlap. Pinhole was set to one Airy unit. A detailed list of recording conditions of all scans used for figures is provided in Supplemental Table 1.

Confocal stacks were imported into ImageJ (Version 1.51h, NIH, http://imagej.nih.gov/ij), contrast and intensity of individual channels were linearly adjusted, and Z-projections of subsets of focal planes or movies were generated. Selected confocal tile scans were stitched using the “pairwise stitching” plug-in for ImageJ (Preibisch et al., 2009). Figures were assembled using InkScape (Version 0.91, www.inkscape.org) and Photoshop (Version 13.0, Adobe CS6).

## Results

### Peripheral targets of catecholaminergic projections

Recently, zebrafish posterior tubercular DA neurons have been shown to innervate peripheral sensory systems, such as the inner ear and lateral line system (Jay et al., 2015; Toro et al., 2015). To provide a detailed description of peripheral DA projections we used Gal4:UAS system transgenic lines for expression of high levels of membrane-tagged GFP in catecholaminergic neurons. We used *Tg(th:Gal4-VP16)*^*m*1233^ and *Tg(UAS:eGFP-CAAX)*^*m*1230^ (Fernandes et al., 2012) double transgenic zebrafish larvae with sufficient membrane-tagged GFP expression in a subset of catecholaminergic neuron groups to visualize both somata and projections throughout the CNS and in the periphery. Where indicated, we used fish that also expressed a synaptophysin-GFP fusion protein from the *Tg(UAS:Syp-GFP, clmc2:GFP)*^*m*1238^ transgene, driven by *Tg(th:Gal4-VP16)*^*m*1233^, to highlight putative synaptic structures in TH-positive neurons. We further took advantage of the CLARITY tissue clearing method (Chung and Deisseroth, 2013), which enabled us to perform anti-TH and anti-GFP immunofluorescence in deep brain regions without the need for permeabilization of fixed larvae by proteinase K digestion, which previously had hindered tracking peripheral fibers due to loss of antigens (Tay et al., 2011). Zebrafish larvae show a rich behavioral repertoire already at 5–6 dpf and start to feed (Budick and O'Malley, 2000). Based on observations that modulatory systems are effective at this stage (Burgess and Granato, 2007; Reinig et al., 2017), we focused our studies on the peripheral projection patterns of the far projecting A11-type DA neurons located in the PT at 6 dpf.

**Figure 1:**
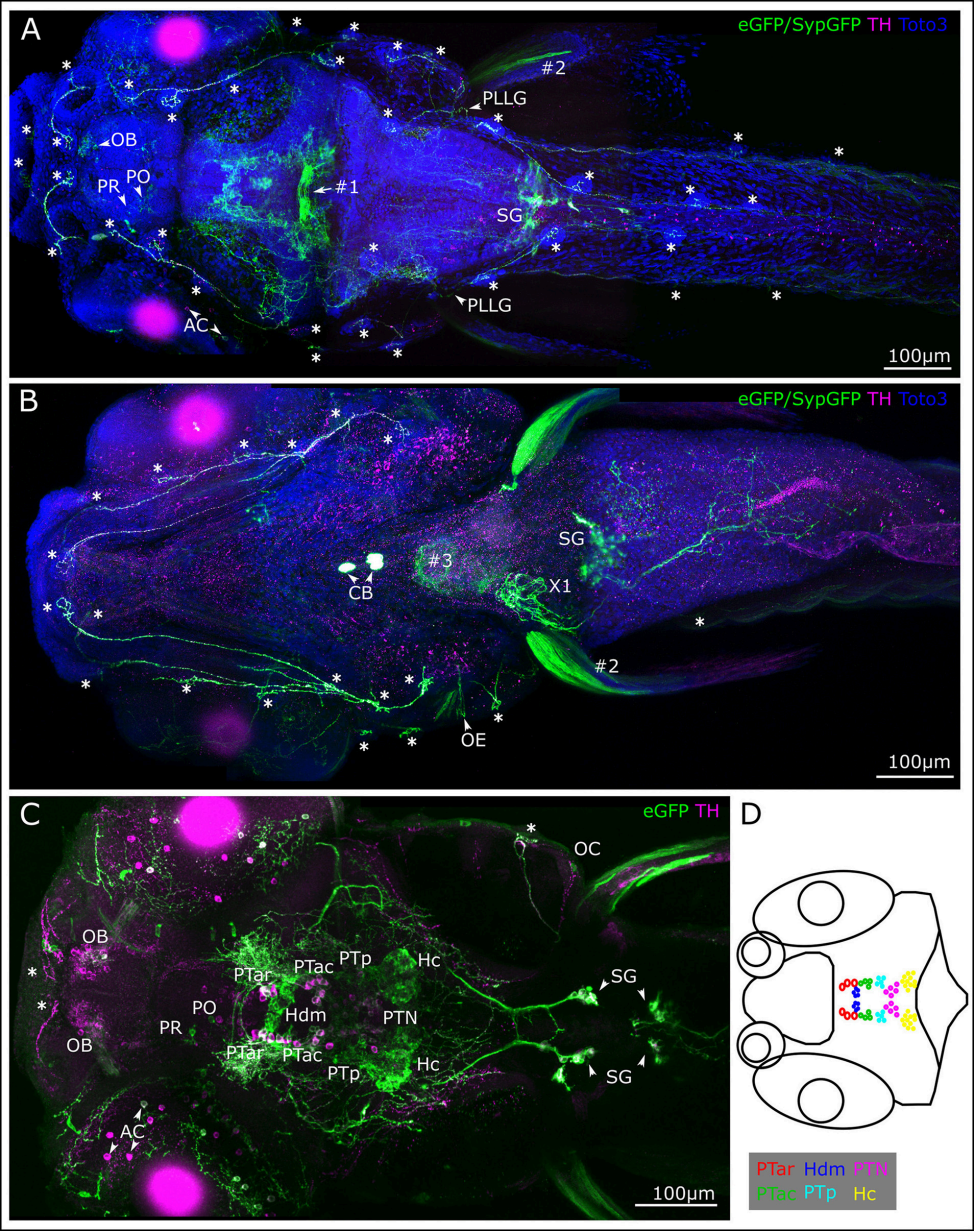
Membrane-tagged GFP expression in 6 dpf *th:Gal4VP16, UAS:GFP-CAAX* larvae identifies peripheral catecholaminergic projections. **(A–C)** Whole mount immunofluorescence detection of GFP and TH in *Tg(th:Gal4-VP16)*^*m*1233^, *Tg(UAS:eGFP-CAAX)*^*m*1230^, *Tg(UAS:SypGFP)*^*m*1238^ transgenic larvae. Images show maximum intensity projections (MIP) from confocal optical sections of larval head and trunk. TH immunoreactivity validates catecholaminergic cells with GFP expression. The immunostain reveals DA cell clusters in the ventral diencephalon/posterior tuberculum (PT) and hypothalamus, in the pretectum (PR), preoptic region (PO) and olfactory bulb (OB), in a subset of amacrine cells (AC) in the retina, as well as noradrenergic hindbrain neurons and sympathetic ganglia (SG). Arrowheads point at structures as indicated by adjacent labels. Central DA projections are labeled throughout the brain and in the spinal cord. Note that GFP expression levels in the transgenic line do not correlate with levels of TH expression, and GFP expression is often found only in a mosaic fashion in a subset of TH positive cells. In the *Tg(th:Gal4-VP16)*^*m*1233^ line, at 6 dpf ectopic GFP expression occurs in a commissure at the border between mes- and rhombencephalon (#1) and in pectoral fin muscles (#2). GFP expression stemming from the heart marker of the *Tg(UAS:Syp-GFP, clmc2:GFP)* transgene is also indicated (#3). No TH-labeling is observed in the ectopically GFP-labeled structures. Cell nuclei were labeled with TOTO-3-Iodide to reveal anatomical landmarks **(A,B)**. **(A)**. Dorsal view MIP (scan from dorsal side, step size 1 μm, total depth of 410 μm, montage of three tiles from rostral part of the whole-mount) with emphasis on peripheral projections going to the lateral line neuromasts (marked with asterisks). PLLG marks posterior lateral line ganglion. **(B)** Ventral view MIP (scan from ventral side, step size 1 μm, total depth of 390 μm), montage of three scanned tiles from rostral part of a whole-mount larva. The ventral scan reveals GFP expression and TH-labeling in peripheral DA projections to the ventrally located lateral line neuromasts (asterisks) and otic epithelium (OE). In addition, the carotid body (CB) CA cells, the SG with their projections, and putative noradrenergic innervation of the atrial region of the heart (X1) are labeled. **(C)** Dorsal view of MIP from optical slices of medial subregion of the whole-mount (montage of two tiles, slices 180–240 of total 507 μm, step size: 1 μm) revealing labeled cell bodies in the depth of the brain. TH immuno-reactive cells are visible in the OB, PR and PO, among those individual cells also express GFP. A subset of TH-immunoreactive AC and their projections express GFP. In the ventral diencephalon, the DA cell clusters in the PT and dorsal hypothalamus are labeled. Double-labeled cell bodies can be observed in the anterior part of the PT in rostral (PTar) and caudal (PTac) sub-clusters, further in the cluster of the posterior PT (PTp) and in the posterior tuberal nucleus (PTN). In the dorsal medial hypothalamus small liquor contacting cells (Hdm) are labeled with GFP as well as in the caudal hypothalamus (Hc). Double labeling can also be observed in the SG. **(D)** Schematic representation of DA cell clusters in the PT and hypothalamus. The cells belonging to the different cell clusters can be identified and distinguished by their location, size, and shape. All scale bars: 100 μm.

Figure 1 introduces the structures labeled in *Tg(th:Gal4-VP16)*^*m*1233^, *Tg(UAS:eGFPCAAX)*^*m*1230^ transgenic fish, including catecholaminergic somata and fibers. Whole mount CLARITY processed larvae were subjected to anti-Tyrosine hydroxylase (TH) and anti-GFP double-immunofluorescence, and counterstained with the nuclear dye TOTO-3 to identify morphological details. Since the *Tg(th:Gal4-VP16)*^*m*1233^ line also drives ectopic expression in non-catecholaminergic cells, we used anti-TH immunofluorescence to validate GFP-expressing catecholaminergic neurons. We documented stained larvae by confocal microscopy, recording stacks from dorsal (Figures 1A,C) and/or ventral (Figure 1B) sides (see Supplemental Table 1 for details on imaging parameters for each embryo recorded). The presentation of the 3D information in 2D images, as well as the balanced visualization of the anti-TH and anti-GFP immunostains proved to be a challenge, and in many images the anti-TH signal is less prominent than the anti-GFP signal. We can only speculate that the membrane-tagged GFP may have been fixed more efficiently in our CLARITY procedure as compared to the cytoplasmic TH. To present as much of the anatomical 3D information as possible, we included as Supplemental Material a confocal image stack with anti-GFP and anti-TH channels (Supplemental Movie 1).

The *Tg(th:Gal4-VP16)*^*m*1233^ line drives Gal4-VP16 expression in all Otp-dependent, DA subgroups in the anterior PT (PTar and PTac, rostral and caudal subclusters, respectively), in the posterior PT (PTp), and in the posterior tuberal nucleus (PTN), which overlaps with TH-immunoreactivity (Figure 1C and Table 1). In the dorsal medial hypothalamus GFP labeling can be observed in small liquor contacting cells (Hdm) and in the caudal hypothalamus (Hc, Figure 1C). The cell clusters in the PT and hypothalamus can be identified and distinguished considering their location along the anterior-posterior axis, their size, and their shape (Figure 1D). The *Tg(th:Gal4-VP16)*^*m*1233^ line sparsely labels olfactory bulb, retinal amacrine, pretectal and preoptic DA neurons, revealing, as judged from anti-TH immunofluorescence, only small subsets of DA neurons in these groups (Figures 1A,C). Like observed for other Gal4:UAS lines in zebrafish, expression within a given neuronal group is often variegated, with expression at different levels and not in all cells of one group (Akitake et al., 2011).

When analyzing catecholaminergic fibers here, we will not comprehensively comment on central projections, which have been described and assigned to specific groups (Tay et al., 2011). Central DA fibers projecting to the periphery exit the brain probably at the level of the hindbrain where they densely arborize. From there, they extend most prominently to the neuromasts of the lateral line (LL) system. Neuromasts consist of sensory hair cells and support cells innervated by afferent and efferent fibers, and are visible in our preparations as distinct structures using TOTO-3-Iodide labeling (Figures 1A,B). The TH-positive projections also contact LL ganglion cells (Figure 1A), the LL primary afferent neurons.

Most hindbrain noradrenergic (NA) neurons of the locus coeruleus and medulla oblongata/area postrema are not expressing GFP in *Tg(th:Gal4-VP16)*^*m*1233^, *Tg(UAS:eGFP-CAAX)*^*m*1230^ transgenic larvae. Therefore, considering also previous findings on CA projections (Kastenhuber et al., 2010; Tay et al., 2011), hindbrain and spinal GFP-positive fibers observed here are likely from DA but not NA neurons. In contrast, peripheral CA neurons in the sympathetic ganglia labeled by GFP expression and TH-immunoreactivity (Figures 1A,C) have previously been shown to be noradrenergic (Holzschuh et al., 2001). GFP-labeled CA fibers, which most likely stem from sympathetic neurons, extend to the gut (Figure 1B) and into the atrial region of the heart (X1). We further observe labeling of the CA carotid body (Figure 1B; Supplemental Movie 1 at level 441 anti-TH and anti-GFP immunoreactive). Finally, ectopic GFP expression can be observed in commissural neurons between the mes- and rhombencephalon (#1, Figure 1A) and in muscle tissue of the pectoral fins (#2, Figure 1B). Ectopic GFP labeling can be distinguished from TH-positive labeling by absence of TH-immunoreactivity. Expression of GFP in the heart stems from the *cardiac myosin light chain 2 (clmc2):GFP* transgene marker used in the transgenesis vector (#3, Figure 1B).

### Retrograde labeling of A11-type DA neurons

**Figure 2:**
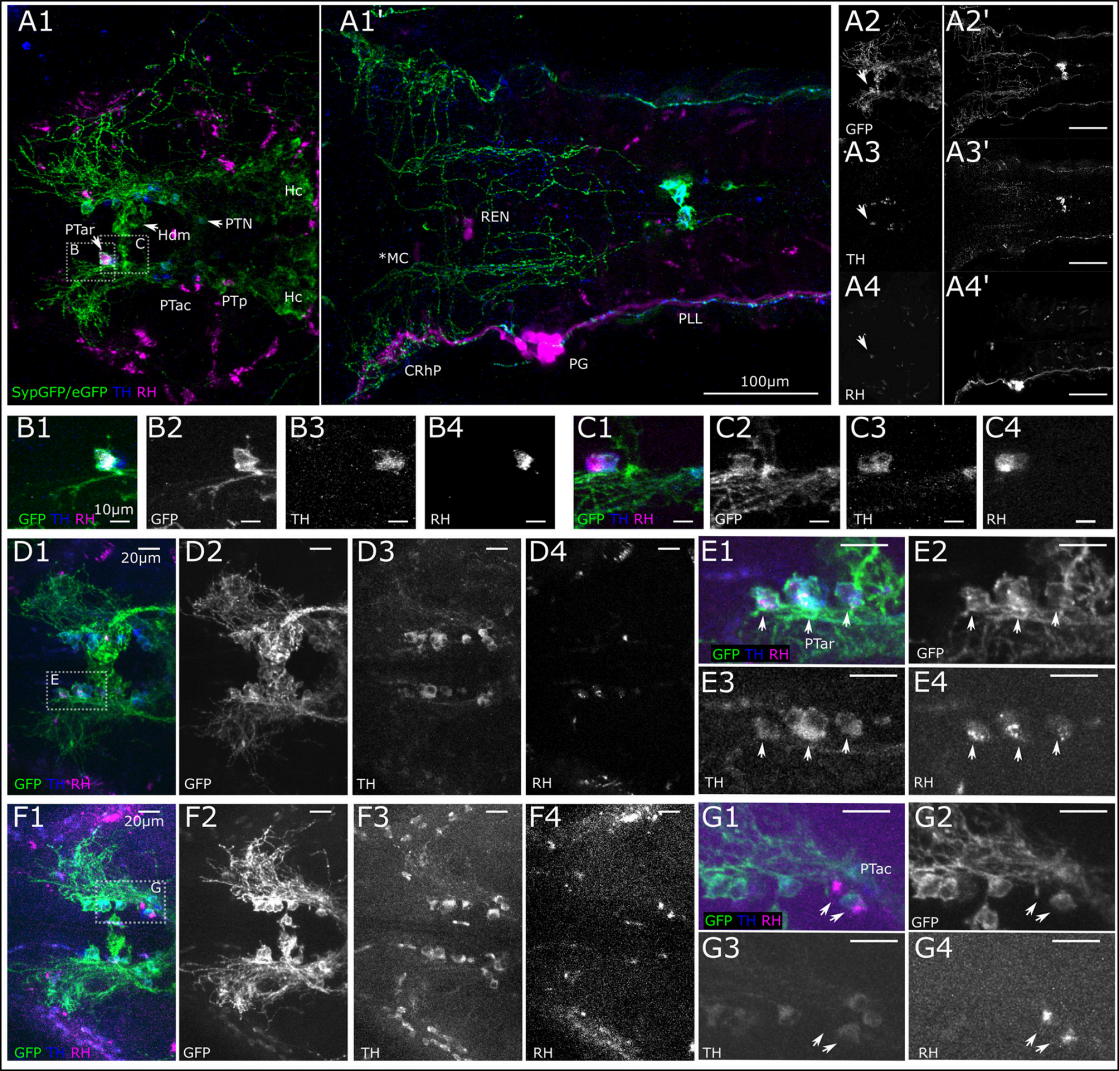
Retrograde labeling of PLL nerve marks DA neurons in the posterior tuberculum. **(A)** Dorsal view montages from two tiles (**A1,A1'**) of MIPs from different substacks of larval head (total depth of 313 μm, step size 1 μm). **(A1)** MIP (slices 185–240) posterior tuberculum (PT) and hypothalamus (H), revealing triple stained cell efferent to the lateral line in the anterior rostral PT (PTar cluster, marked with arrow). Further DA cell clusters: anterior caudal PT (PTac), medial dorsal H (Hdm), posterior PT (PTp), posterior tuberal nucleus (PTN) and caudal H (Hc). SypGFP/eGFP-CAAX driven by *th:Gal4-VP16*: green, TH immunoreactivity: blue. **(A1')** MIP (slices 80–150) showing backfill of lateral line neurons. Rhodamine dextran (magenta) was injected into the posterior lateral line nerve (PLL); afferent neurons are labeled along the nerve, in the posterior lateral line ganglion (PG) and central rhombencephalic projection (CRhP). Efferent neurons are labeled rhombencephalic efferent neurons (REN), asterisk marks lateral dendrite of the Mauthner cell (MC), which has also faintly taken up dye. SypGFP/eGFP-CAAX expression **(A2,A2')**. TH-immunoreactivity **(A3,A3')**. Retrograde Rhodamine dextran labeling **(A4,A4')**. All scale bars: 100 μm. **(B)**. Magnification of region as indicated in **(A1)** with Rhodamine dextran backfilled, TH and GFP immunoreactive DA PTar cell. MIP of substack (total depth of 16 μm). **(C)**. Substack from MIP of region indicated in **(A1)**, ventrally of **(B)** (total depth 16 μm) revealing two more rhodamine labeled cells in the PTar cluster. Scale bars **(B,C)**: 10 μm. **(D)**. Dorsal view of MIP of DA-cell clusters in PT with three Rhodamine dextran backfilled, TH and GFP immunoreactive DA cells (step size: 1 μm, total depth of 35 μm). **(E)**. Magnification of region indicated in **(D1)**. MIP of PTar cell bodies (total depth of 17 μm). **(F)**. Dorsal view (MIP) of DA-cell clusters in PT with two Rhodamine dextran backfilled, TH and GFP immunoreactive DA PTac cells (step size: 1 μm, total depth of 35 μm). **(G)**. Magnification of region indicated in **(F1)**. MIP of PTac cell bodies (total depth of 13 μm). Scale bars **(D–G)**: 20 μm. Pseudo coloring and sub-panels in **(B–G)** as described in **(A)**, all panels labeled GFP show expression of SypGFP/eGFP-CAAX detected by anti GFP immunofluorescence. Step size for all: 1 μm.

We next wanted to trace the CA fibers contacting the lateral line neuromasts to their corresponding somata, in order to resolve whether they are dopaminergic (TH-immunoreactive groups previously shown to coexpress dopamine transporter) or potentially noradrenergic (which coexpress Dopamine beta hydroxylase; Holzschuh et al., 2001). Therefore, we deposited rhodamine dextran into the PLL nerve at the location of a neuromast, and were able to retrogradely trace the projections to DA somata in the PT (Figure 2). We injected approximately 30 larvae at 5 dpf with rhodamine dextran into the PLL nerve. Successful injection was confirmed by observing labeling of afferent neurons at 6 dpf *in vivo* in the LL ganglion. In five larvae we observed labeled neurons in the PT using the CLARITY method (Table 4), specifically in the PTar and PTac clusters. We also observed labeled LL afferent neurons in the ganglion and rhombencephalic LL efferent neurons (Figure 2A'). In most cases, we observed dextran labeling in PTar neurons, which overlapped with GFP expression and TH-immunoreactivity (Figures 2A–E). In one larva, we observed rhodamine labeling of PTac neurons (Figures 2F,G). We conclude that the rostral posterior tubercular subgroups PTar and PTac are the predominant source of efferent CA innervation of neuromasts, which suggests that these fibers are indeed dopaminergic. Therefore, in the following we will refer to the CA fibers projecting to the lateral line as posterior tubercular DA fibers. We only directly showed the connection between PT DA neurons and the peripheral fibers for the posterior lateral line, but deem it unlikely that the source of CA innervation is different for the anterior lateral line.

**Table 4:** Retrogradely labeled DA neurons in the posterior tuberculum (Results).

**Larva Nr**.	**PTar**	**PTac**	**Figures**
1	2	0	9A–C
2	≥3	0	9D–E
3	0	2	9F–G
4	1	0	n.s.
5	1	0	n.s.

*Approximately 30 larvae were injected with rhodamine dextran into the posterior lateral line nerve at a neuromast. Dye uptake was confirmed in vivo, by observing dye filling of lateral line afferent neurons. Filling of DA efferent neurons was inspected under the confocal microscope after tissue clearing with CLARITY. (n.s., data not shown)*.

### Innervation of the lateral line system

**Figure 3:**
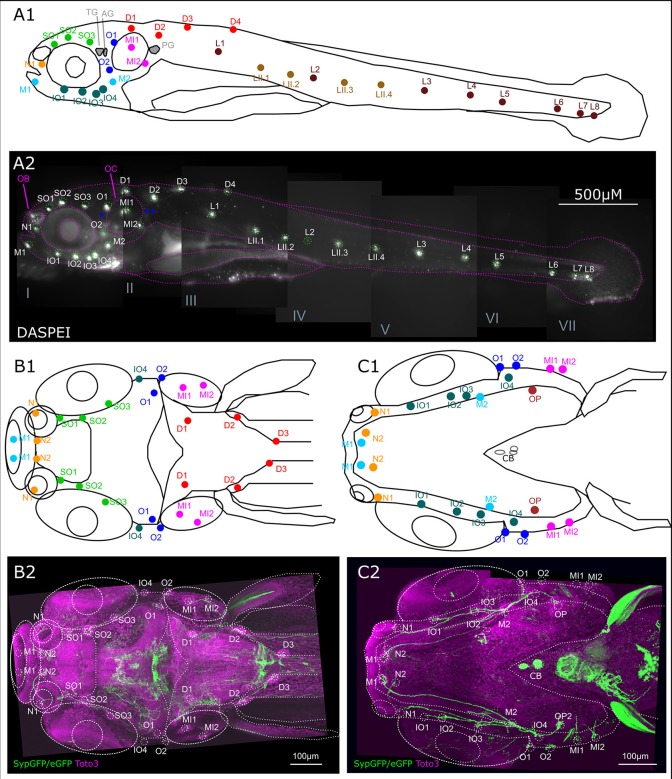
Overview of lateral line system. **(A)** Lateral view of 6 dpf larva with neuromasts of anterior and posterior lateral line system. Schematic drawing of neuromasts viewed from lateral. **(A1)** Approximate location of trigeminal ganglion (TG), anterior (AG) and posterior lateral line ganglion (PG): gray. Colors and abbreviations of head neuromasts described below **(B,C)**, lateral branch of posterior lateral line (maroon): L1–L8, secondary lateral branch (light brown): LII.1–LII4. **(A2)** Lateral view of montage of seven optical stacks taken with fluorescence microscope of live 6 dpf larva stained with DASPEI *in vivo* to visualize neuromasts. Schematic as shown in **(A1)** overlaid in magenta, neuromasts marked in green. Location of trigeminal and lateral line ganglia marked with blue asterisks. Landmark structures labeled: olfactory bulb (OB) also slightly stained, otic capsule (OC). Depth of stacks: I: 62 μm, II: 250 μm, III: 82 μm, IV: 134 μm, V: 160 μm, VI: 110 μm, VII: 34 μm, step size: 2 μm. **(B)**. Dorsal view of 6 dpf larval head with neuromasts of the anterior lateral line system as well as dorsal and medial branch of posterior lateral line system. **(B1)** Schematic drawing of anterior and dorsal lateral line neuromasts relative to anatomical landmarks. Dorsal branch (red) D1–D3 and medial branch (magenta) MI1 and MI2, all with cell bodies of their afferent neurons in the posterior/medial-posterior lateral line ganglion. Otic branch (blue) O1 and O2; Infraorbital branch (dark green) IO4; supraorbital branch (light green) SO1–SO3; nasal branch (orange) N1 and N2; and mandibular branch (cyan) M1, all with cell bodies of their afferent neurons in the anterior lateral line ganglion. **(B2)** Dorsal view of Z-MIP of montage of two tiles of rostral part of a whole mount of entire larva (total depth: 477 μm, step size: 1 μm) with overlaid schematic as shown in **(B1)** eGFP-CAAX/SypGFP expression driven by th:Gal4-VP16 visualized by immunofluorescence in green, TOTO-3 labeling of cell nuclei in magenta. **(C)** Ventral view of 6 dpf larval head with neuromasts of the anterior and otic lateral line. **(C1)** Schematic drawing relative to anatomical landmarks. MI1–MI2 (magenta), O1–O2 (dark blue), with cell bodies of innervating afferents in the posterior/medial-posterior lateral line ganglion. Opercular neuromast (dark red): OP, infraorbital branch (dark green): IO1–IO4, N1–N2 (orange), mandibular branch (cyan): M1–M2, with cell bodies of innervating afferents in the anterior lateral line ganglion. **(C2)** Ventral view of Z-MIP of montage of two tiles of rostral part of a whole mount of entire larva (total depth: 434 μm, step size: 1 μm) with overlaid schematic as shown in **(C1)**. Pseudo colors as in **(B)**.

The efferent DA fibers observed in 6 dpf larvae course along the nerves of the lateral line in parallel with the afferent fibers of the anterior and posterior lateral line (ALL and PLL, respectively). They send collaterals to the neuromasts which develop in a stereotypical pattern and are individually identifiable at larval stages (Raible and Kruse, 2000; Haehnel et al., 2012). These projections represent the most prominent DA fibers in the periphery. Figure 3 presents the locations of neuromasts in the ALL and PLL system at 6 dpf. The afferent neurons of the PLL nerve alongside the afferent neurons that innervate the dorsal neuromasts and the secondary line of PLL neuromasts are located in the PLL ganglion caudal of the otic capsule. Slightly rostrally to this, the ganglion cells that innervate the medial neuromasts are located and sometimes referred to as the medial LL ganglion. A lateral view of the entire larva at 6 dpf with an *in vivo* DASPEI stain, which labels the hair cells, shows the locations of the ALL neuromasts on the head and the PLL neuromasts along the trunk (Figure 3A).

In the head, neuromasts develop along ALL nerve branches. The afferent neurons are located in the ALL ganglion, next to the trigeminal ganglion. The efferent catecholaminergic fibers, presumably originating from DA posterior tubercular neurons, follow these branches and send collaterals to each neuromast of the ALL (Figures 3B,C).

**Figure 4:**
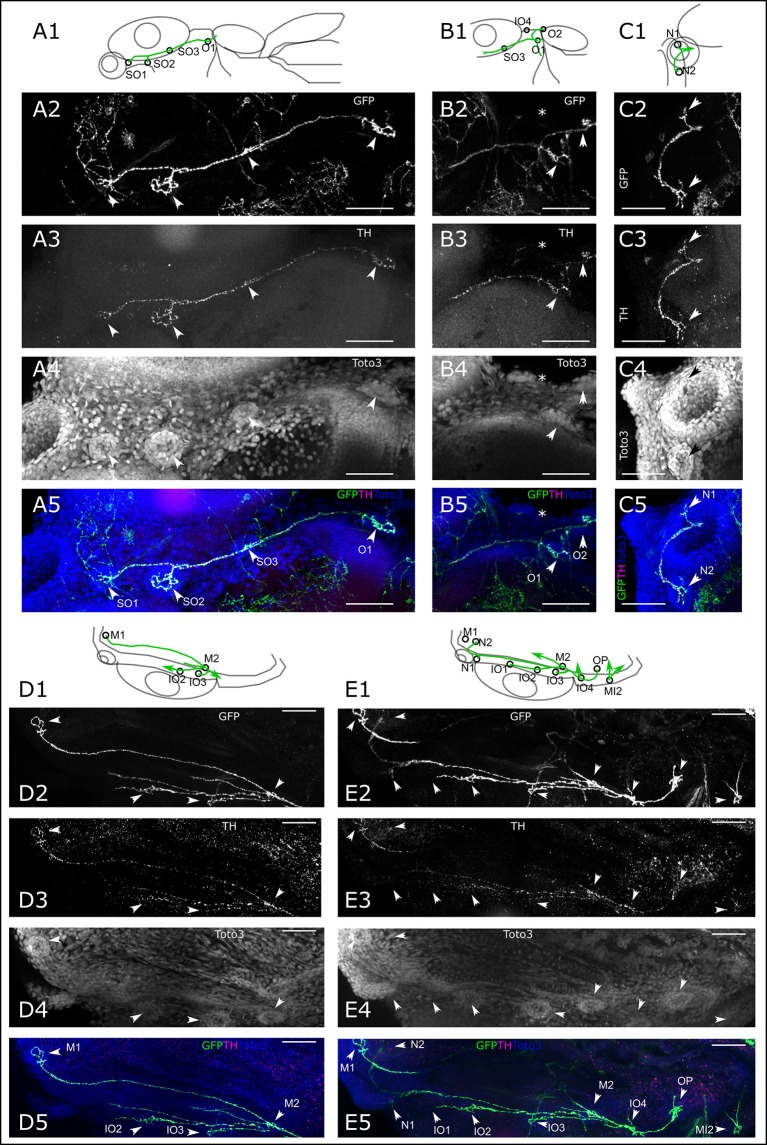
Dopaminergic innervation of the anterior lateral line neuromasts. **(A–E)** Panels 1: schematic showing location of projections (green) and neuromasts (circles) relative to anatomical landmarks. Panels 2 and 5 (green): th:Gal4 driven eGFP-CAAX and SypGFP (GFP) expression visualized by immunofluorescence. Panels 3 and 5 (magenta): anti-TH immunofluorescene. Panels 4 and 5 (blue): TOTO-3 labeling of cell nuclei. MIP of confocal stacks, step size **(A–E)** 1 μm, scale bars: 50 μm. **(A)**. Dorsal view of right half of larval head (total depth of 135 μm) containing neuromasts (NM) of the otic (O) and supraorbital branch (SO). Arrowheads point to NM SO1–SO3 and O1. **(B)**. Right lateral part of head, dorsal view of DA projections to NM SO3 and O1 and O2. Also visible, ventrally located NM IO4 (asterisk). Total depth of 122 μm. **(C)**. Right frontal part of head, dorsal view of DA projection to nasal NM (N), total depth of 122 μm. **(D)**. Right lateral part of head ventral view of DA projection to NM of the infraorbital branch (IO2 and IO3) and mandibular NM (M), total depth of 91 μm. **(E)**. Right lateral part of head ventral view of DA projection to NM of the infraorbital branch (IO), mandibular (M), nasal (N), and opercular branch (OP). Also visible, the medial NM MI2. Total depth of 226 μm.

We next took a closer look at the individual branches of the LL system to determine whether all visible neuromasts receive DA innervation at 6 dpf. We first looked at the ALL neuromasts. All supraorbital neuromasts, as well as the medial neuromasts are innervated by DA fibers of one or a few axons (Figure 4A). DA fibers innervate both otic neuromasts (Figure 4B). In the rostral head, we see DA fibers projecting to both nasal neuromasts (Figure 4C). A ventral z-stack reveals innervation of the mandibular and infraorbital neuromasts (Figure 4D), as well as opercular and otic neuromasts (Figure 4E). The same z-stack also shows that the innervation of the nasal neuromasts extends from the infraorbital branch.

**Figure 5:**
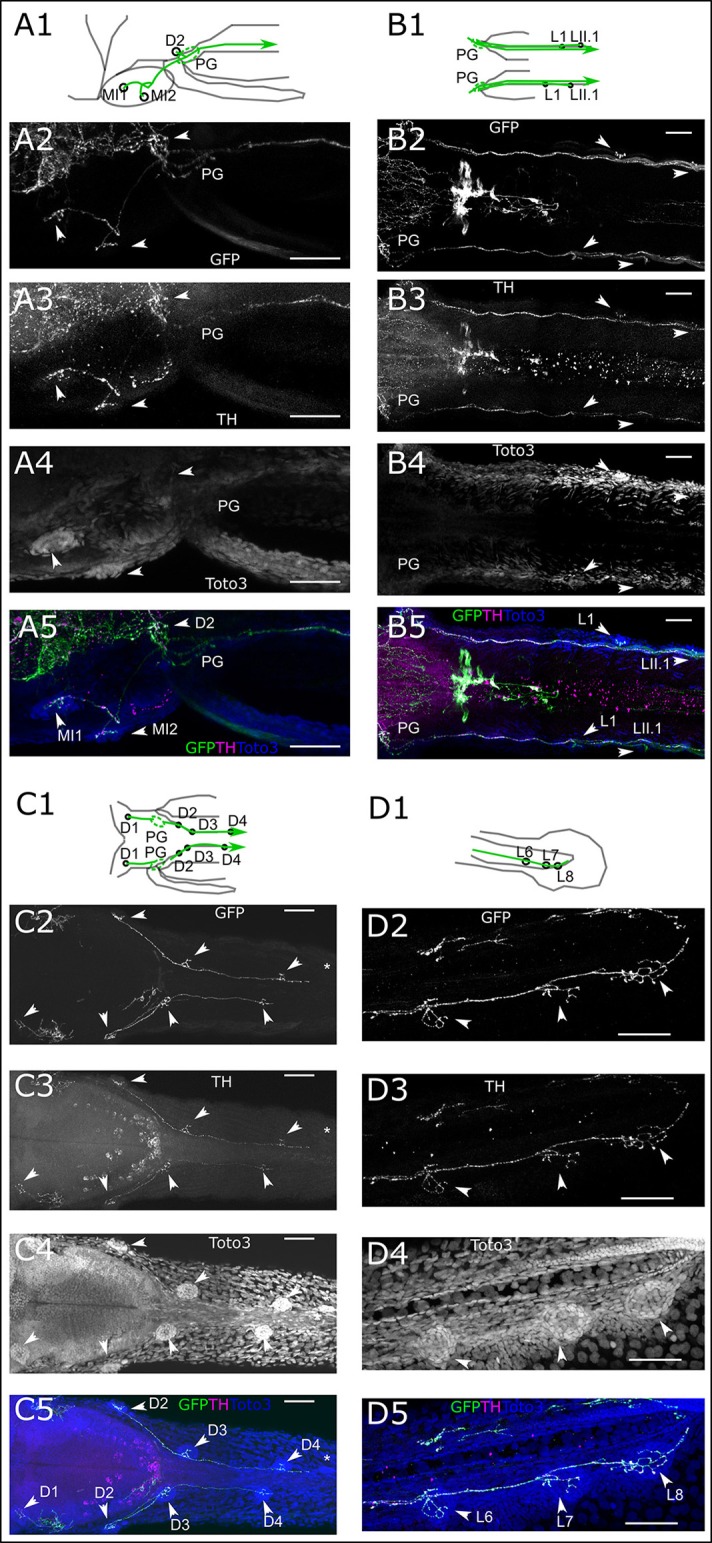
Dopaminergic innervation of the posterior lateral line neuromasts. **(A–D)** Panels 1: schematic showing location of projections (green) and neuromasts (circles) relative to anatomical landmarks. Panels 2 and 5 (green): th:Gal4 driven eGFP-CAAX and SypGFP (GFP) expression visualized by immunofluorescene. Panels 3 and 5 (magenta): anti-TH immunofluorescene. Panels 4 and 5 (blue): TOTO-3 labeling of cell nuclei. MIP of confocal stacks, step size **(A–D)** 1 μm, scale bars: 50 μm. Arrowheads point at neuromasts. **(A)**. Left lateral part of head dorsal view of DA projections to NM of the medial branch (MI) and dorsal NM D2, as well as projection going through the posterior lateral line ganglion (PG) and toward the posterior lateral line nerve. Total depth of 175 μm. **(B)**. Dorsal view of DA projection to NM L1 and LII.1. Total depth of 130 μm. **(C)**. Dorsal view of DA projection to neuromasts of the dorsal branch **(D)** of the posterior lateral line. Asterisk indicates putatively developing neuromast without innervation by GFP labeled fibers. Total depth of 60 μm. **(D)**. Lateral view of DA projection to tail NM of the lateral branch (L) of the posterior lateral line. Total depth of 25 μm.

In the trunk region, we see DA fibers projecting to the PLL ganglion, and rostral from the ganglion fibers extending to the medial neuromasts, as well as caudally projecting fibers along the PLL nerve (Figure 5A). The fibers coursing along the PLL nerve appear to contact each neuromast of the primary and secondary PLL branch, located laterally on the trunk (Figure 5B). On the dorsal trunk, we observe DA fibers projecting to four dorsal neuromasts on each side, while the TOTO-3 label reveals a fifth developing neuromast, which does not yet receive DA innervation (Figure 5C). The lateral DA projections run all the way to the tail tip of the larva, where they contact the tail PLL neuromasts (Figure 5D).

Table 5 summarizes the number of scans in which we observed GFP- and TH-immunoreactive axons at the lateral line neuromasts, for each neuromast in 6 dpf old larvae. The data reveal that at this stage, lateral line organs receive dopaminergic efferent innervation shortly after they develop.

**Table 5:** Neuromasts observed to receive innervations from DA-projection (Results).

**Larvae analyzed for**	**Lateral line organs analyzed**												
**ANTERIOR LATERAL LINE**													
SO	N1	N2	M1	M2	SO1	SO2	SO3	IO1	IO2	IO3	IO4	O1	O2
GFP pos.	9	9	7	4	11	12	12	4	4	4	4	12	12
TH ir	9	9	7	4	10	11	12	4	4	4	4	12	12
**POSTERIOR LATERAL LINE (MEDIAL AND DORSAL BRANCHES)**													
SO	MI1	MI2	OP	D1	D2	D3	D4	D5					
GFP pos.	11	11	5	11	14	8	8	5					
TH ir	11	11	5	10	13	8	7	4					
**POSTERIOR LATERAL LINE (LATERAL BRANCHES)**													
SO	L1	L2	L3	L4	L5	L6	L7	L8	LII.1	LII.2	LII.3	LII.4	
GFP pos.	8	7	4	5	3	4	2	2	5	3	2	1	
TH ir	8	7	5	5	3	4	2	2	6	3	2	1	

*A total number of 32 scans were analyzed and checked for GFP positive innervation (GFP pos.) and TH-immunoreactivity (TH ir). Scans were made from different body regions and with varying orientation of the larvae. 19 scans were made with th:Gal4 larvae expressing UAS:eGFP, 10 th:Gal4 larvae expressed UAS:eGFP-CAAX and additionally UAS:SypGFP, and 3 th:Gal4 larvae expressed only UAS:SypGFP. Discrepancies between TH-immuno-labeling and GFP label likely stem from variegated GFP expression*.

### Synapse-like structures

**Figure 6:**
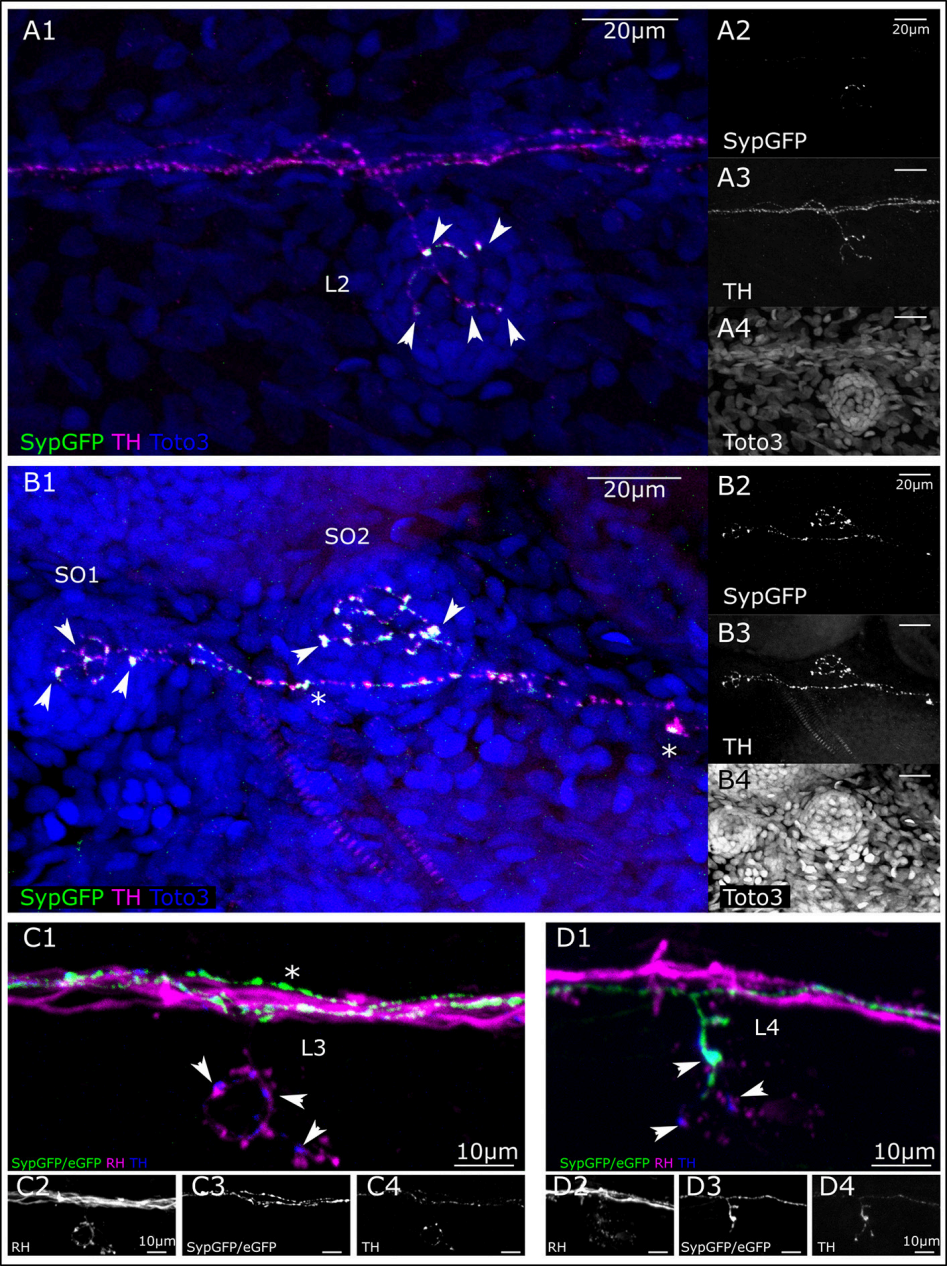
Putative pre-synaptic structures in dopaminergic fibers innervating lateral line neuromasts. **(A)** Lateral view (MIP) of L2 NM immunofluorescence labeled in transgenic *th:Gal4, UAS:SypGFP* 6 dpf larva to highlight synaptic structures. **(A1)** Merged image of channels shown in **(A2–A4)**. Total depth of 47 μm. SypGFP green and in **(A2)**, anti-TH magenta and in **(A3)**, TOTO-3 blue and in **(A4)**. Arrowheads point to putative synaptic structures. **(B)** Dorsal view (MIP) of SO1 and SO2 NM in the anterior lateral line, immunofluorescence labeled in transgenic *th:Gal4, UAS:SypGFP* 6 dpf larva. **(B1)** Merged image of channels shown in **(B2–B4)** Total depth of 59 μm. SypGFP green and in **(B2)**, anti-TH magenta and in **(B3)**, TOTO-3 blue and in **(B4)**. Arrowheads point to putative synaptic structures. Asterisks: Putative axonal transport vesicles containing synaptophysin. **(C)** Lateral view (MIP) of L3 NM, immunofluorescence labeled in transgenic *th:Gal4, UAS:SypGFP, UAS:EGFP-CAAX* larva, with retrograde fill from lateral line nerve with rhodamine dextran (RH) dye to trace the afferent neurons. **(C1)** Merged image of channels shown in **(C2–C4)**. Afferent neurons labeled with rhodamine dextran shown in magenta and in **(C2)**, SypGFP/EGFP-CAAX green and in **(C3)**, anti-TH blue and in **(C4)**, total depth of: 34 μm. Arrow heads point to putative synapses between the afferent neuron (magenta) and the efferent DA projection (blue, anti-TH immunoreactivity). Asterisk indicates axon of DA projection passing by the L3 neuromast. **(D)** Lateral view of L4 NM (MIP, same fish as in **C**). **(D1)** Merged image of channels shown in **(D2–D4)**, total depth of 37 μm. Arrowheads point to putative synapses between the afferent neurons (magenta) and the efferent DA projection labeled with GFP (green, and **D3**) and anti-TH (blue, and **D4**). The projection labeled by the expressed GFP enters NM L4. Step size for **(A–D)** 1 μm. Scale bars **(A,B)** 20 μm, **(C,D)** 10 μm.

We used *th:Gal4* driven Synaptophysin-GFP expression from *Tg(UAS:Syp-GFP, clmc2:GFP)*^*m*1238^ to identify putative synapses in CA fibers. In larvae that we screened for the expression of Syp-GFP, but absence of eGFP-CAAX, we see expression in varicosities at the bases of cells in the center of the lateral line neuromasts, which are presumably hair cells (Figures 6A,B). We also observe TH-immunoreactivity all along the DA axons in the PLL nerve, whereas Syp-GFP expression appears only in varicosities, indicating putative pre-synaptic structures in a trunk neuromast (Figure 6A). We can identify at least three individual DA fibers along the PLL nerve. One of those appears to send off a collateral to the specific L2 neuromast. In two head neuromasts (SO1 and SO2), Syp-GFP expression was likewise detected in varicosities around putative hair cells. However, we also observe spots of GFP expression along the axon, suggesting vesicular transport of Syp-GFP (Figure 6B).

We next deposited rhodamine dextran in a caudal part of the PLL nerve to retrogradely label afferent LL neurons (Figures 6C,D). Figure 6C shows a trunk L3 neuromast that is innervated by a TH-immunoreactive efferent fiber, but by-passed by two other GFP expressing DA fibers. The presence of non-GFP expressing but TH positive fibers illustrates the variegated expression of GFP driven by the *th:Gal4* line due to transcriptional silencing (Akitake et al., 2011). However, we observe TH-immunoreactive varicosities in close proximity to swellings on the dextran-labeled afferent neurons, indicating that DA release may occur close to the hair cell/sensory afferent ribbon synapse. In the same animal, at least one of the GFP-expressing DA fibers contacts the L4 neuromast located directly caudal of the one shown in Figure 6C, and we observe varicosities indicative of putative synapses (Figure 6D). Figures 6C,D also reveal that three or more DA efferents contribute to the lateral line nerve, and that distinct DA efferent fibers appear to innervate different subsets of neuromasts in the trunk and tail.

### Innervation of sensory ganglia

**Figure 7:**
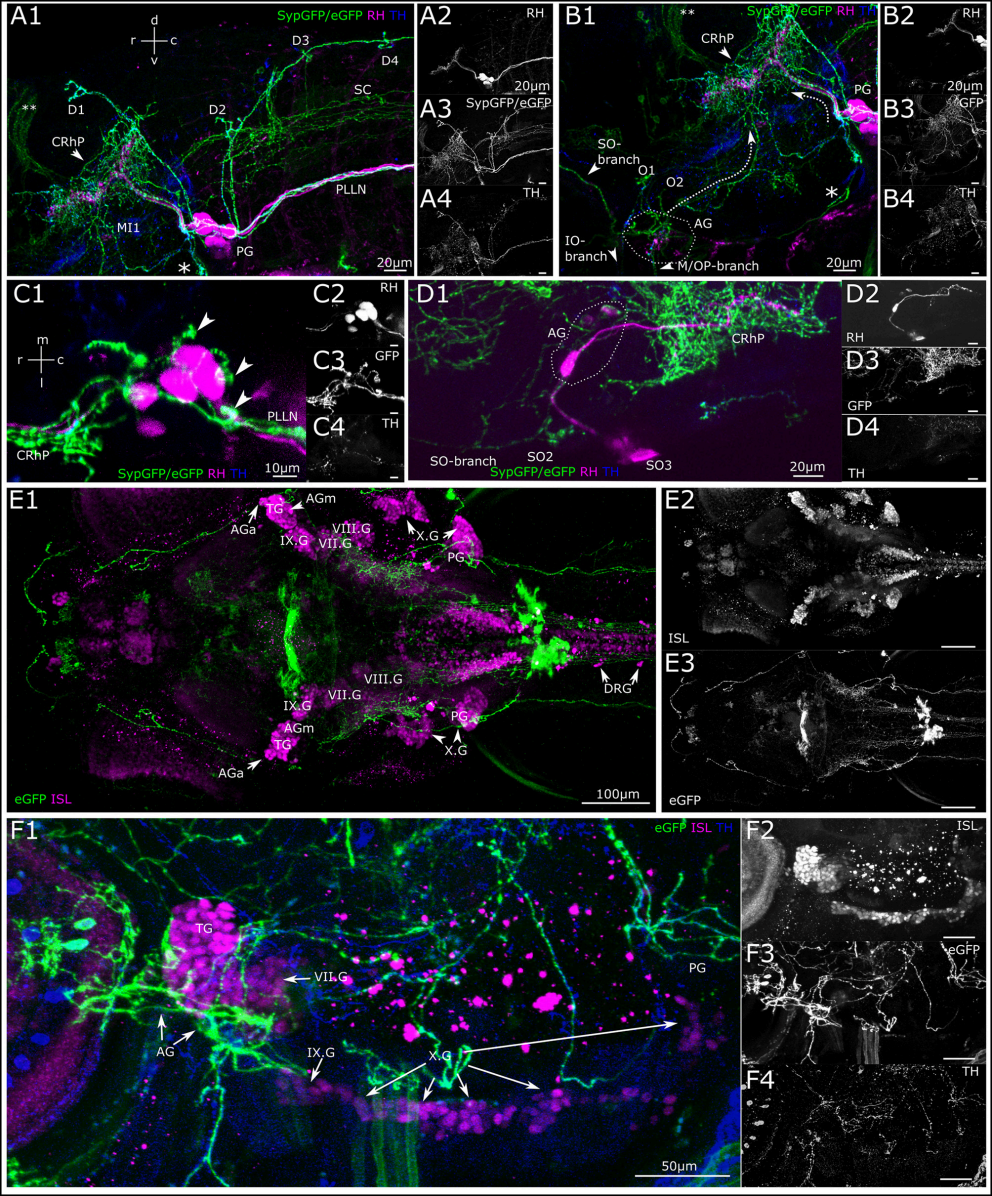
Dopaminergic projections to lateral line ganglia and other cranial ganglia. **(A)** Lateral view (MIP) of the region around the posterior lateral line ganglion (PG). Step size: 1 μm, total depth of: 81 μm. Primary afferent lateral line neurons retrogradely labeled with rhodamine dextran (**A2**, magenta in **A1**) by dye injection into the posterior lateral line nerve (PLLN) at a neuromast. A number of afferent neurons took up dye, which have cell bodies residing in the PG and project centrally into the rhombencephalon (CRhP, arrowhead). SypGFP/eGFP-CAAX labeling driven by th:Gal4-VP16 (**A3**, green in **A1**). DA-axons project through the PG and along the PLLN as well as to the dorsal (D) and otic (O) neuromasts. Ventrally, the projection branches toward the vagus ganglia (asterisk). Dorsally, the DA-projection runs parallel with the CRhP of the afferent neurons. Central DA-projections are present in the spinal cord (SC). Double asterisks: ectopically labeled commissure. TH-immunoreactivity of efferent DA-projections (**A4**, blue in **A1**). **(B)** Lateral view (MIP) of region around the anterior lateral line ganglion (AG), step size: 1 μm, total depth: 81 μm. Rhodamine was injected into the PLLN labeling afferent neurons of the posterior lateral line (**B2**, magenta in **B1**), but not of the anterior lateral line. DA-axons projecting through the AG labeled with th:Gal4-VP16 driven SypGFP/eGFP-CAAX (**B3**, green in **B1**), and projecting rostrally parallel to the infraorbital (IO) and supraorbital (SO) branch, as well as caudally (dotted arrow) toward the CRhP (arrow head) and ventrally to the mandibular (M) and opercular (OP) neuromasts. Asterisk marks the branch of the DA projection coming from the PG and running along the region of the vagus ganglia. Double asterisk: ectopically labeled commissure. TH-immunoreactivity labeling DA projections (**B4**, blue in **B1**). Orientation for **A, B** indicated in **(A1)**. **(C)** Dorsal view (MIP) of PG. Pseudo-colors and channels as in **(B)**. Step size: 1 μm, total depth: 59 μm. DA projections running through the PG. Arrowheads in C1 pointing to DA fibers projecting dorsally along the lateral line nerve branches toward the dorsal neuromasts. Rostrally, DA-projections run parallel with afferent neurons to CRhP. **(D)** Dorsal view (MIP) of region around AG. Rhodamine dextran (magenta in **D1**) was injected into the MI1/MI2 branch of the anterior lateral line and labels few afferent anterior lateral line neurons, which centrally project into the hindbrain, with efferent DA projection arborizing around the CRhP (green in **D1**). Step size: 1 μm, total depth: 155 μm. Orientation for **(C,D)**, indicated in **(C1)**. **(E)**. Dorsal view (MIP) of whole mount, montage of two stitched tiles with larval head. Step size: 1 μm, total depth: 476 μm. Cell bodies of sensory afferent neurons are labeled with primary anti-Islet1/Islet2 (ISL), and secondary Alexa555 antibodies (**E2**, magenta in **E1**) in the cranial and dorsal root ganglia (AGa, anterior part of anterior lateral line ganglion; AGm, medial part of AG; TG, trigeminal ganglion; PG, posterior lateral line ganglion; DRG, dorsal root ganglia; VII.G, facial ganglion; VIII.G, statoacoustic ganglion; IX.G, glossopharyngeal ganglion; X.G, vagus ganglia). Th:Gal4 driven eGFP-CAAX labeling of DA-projections **(E3)**. **(F)** Lateral view (MIP) of region with AG and PG. (step size, 3.63 μm, depth 137.9 μm). Pseudo coloring and channels as in **(E)**. TH-immunoreactivity, **(F4)** and blue in **(F1)**. DA projection contacting the AG and TG, and further projecting to the vagal ganglia.

Besides the above-described projections to the neuromasts of the lateral line, we observe DA projections invading the sensory ganglia (Figure 7). By depositing rhodamine dextran into the PLL nerve, we aimed to investigate DA projections to the PLL ganglion and observe DA fibers relative to the PLL afferent somata (Figure 7A). We observe DA projections crossing through the posterior ganglion, in between afferent somata, and from there running in parallel with afferent fibers in the PLL nerve as well as along with the afferent branches toward the dorsal and otic neuromasts. DA fibers also run in parallel with afferent fibers in the rhombencephalon, where DA fibers form extensive arborizations around the LL projection field in the MON. Further DA fibers appear to branch off ventrally from the PLL ganglion toward the vagal ganglia. We also see DA fibers running in parallel with the ALL nerve branches and into the region of the ALL ganglion, which can be tracked back into the rhombencephalon (Figure 7B); however we cannot completely exclude crossing sympathetic CA fibers. A dorsal view of a retrogradely labeled subset of PLL afferent neurons shows that DA fibers course between the somata (Figure 7C). Figure 7D shows retrogradely labeled ALL afferent neurons with somata in the ALL ganglion and their central projection field in the rhombencephalon, as well as parallel innervation by DA fibers, which extensively arborize around the afferent projection field (CRhP in Figures 7C,D) in the MON.

To investigate potential posterior tubercular DA fibers relative to other sensory ganglia we used an antibody against the Islet1/2 proteins, which are expressed in all sensory ganglia in larval zebrafish (Figure 7E). The lateral view (Figure 7F) shows the trigeminal ganglion with strong Islet-immunoreactivity and the ALL and PLL ganglia with faint Islet-immunoreactivity targeted by catecholaminergic fibers. At least some of these fibers appear to be the same dopaminergic fibers running in parallel with the lateral line afferent fibers in the LL nerves. However, we cannot exclude that some of the fibers are catecholaminergic of sympathetic origin. There appears to be a higher density of CA fibers running through the ALL ganglion compared to the trigeminal ganglion, and the projection further extends through the glossopharyngeal ganglion (Supplemental Figure 1A). Islet-immunoreactivity in the PLL ganglion is weak, but can be observed in the main PLL ganglion and the medial LL ganglion (Supplemental Figure 1B).

### Innervation of trigeminal, auditory, and vestibular system

**Figure 8:**
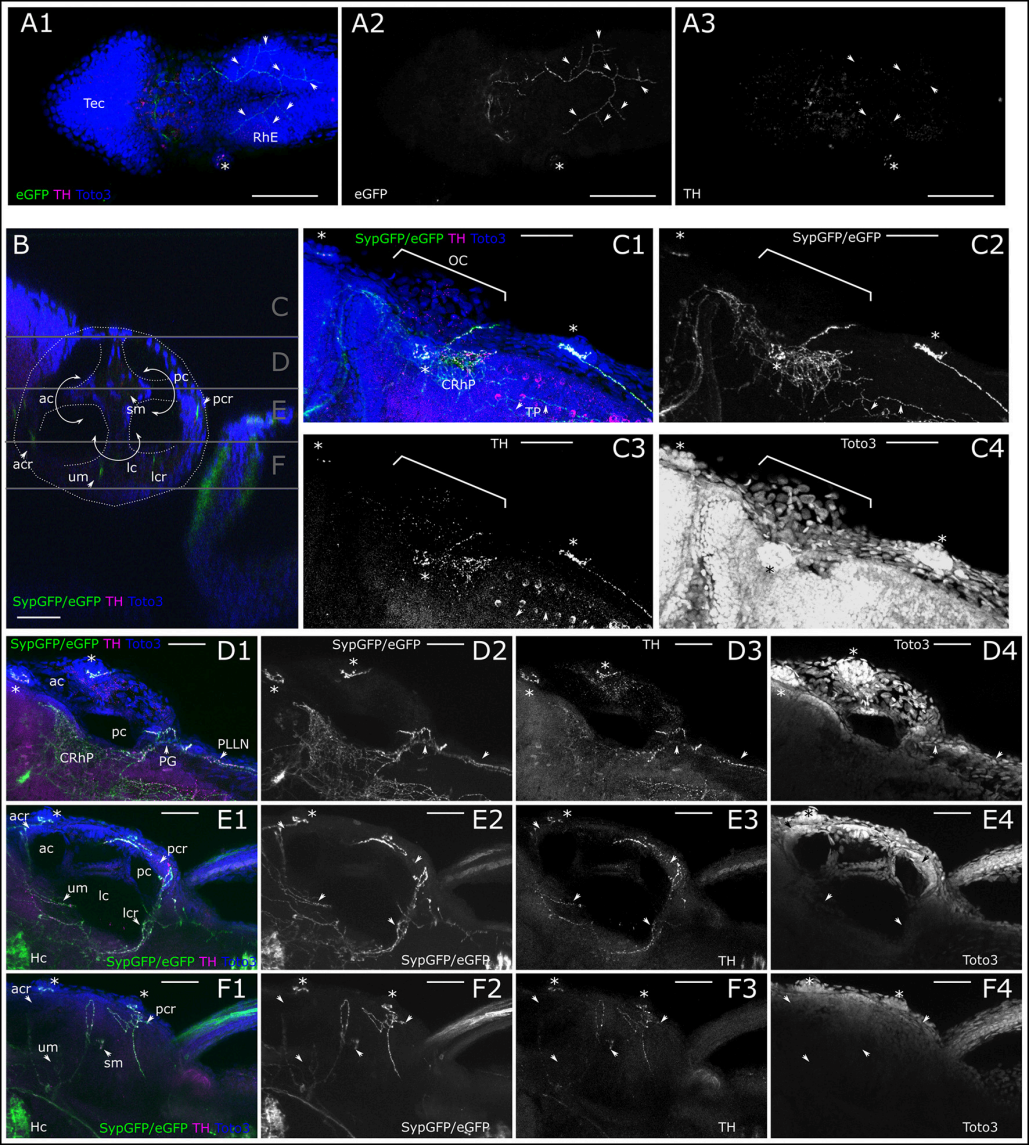
Dopaminergic innervation of trigeminal nerve endings and inner ear epithelium. **(A)** MIP of dorsal part of larval head (Slices 16–40 of 507 μm stack, step size: 1 μm) with the tectum (Tec) and rhombencephalon (RhE) showing potential CA arborizations, which could represent parallel innervation of trigeminal free nerve endings in the skin. Asterisk marks supraorbital neuromast. Arrowheads point to branches of the skin innervation labeled with th:Gal4 driven eGFP-CAAX (TOTO-3: **A2** and blue in **A1**) and faint TH-immunoreactivity (**A3**, magenta in **A1**). **(B)** Sagittal section from XZ-orthoslice at 173 μm of z-stack (dorsal scan, step size: 1 μm, total depth: 462 μm, total width 509 μm) showing the developing inner ear outlined in white with the developing canals of the labyrinth, divided by epithelial pillars (white solid arrows) and relative location of the sensory epithelia with DA innervation (ac, anterior canal; acr, anterior crista; lc, lateral canal; lcr, lateral crista; pc, posterior canal; pcr, posterior crista; sm, saccular macula; um, utricular macula; Whitfield et al., 2002). Gray horizontal lines indicate sections of Z-MIP shown in **(C–F)**. **(C)**. Z-MIP of otic slice stack “C” indicated in **(B)** (slice 1–125 of 462, step size: 1 μm) with otic capsule (OC). Asterisks mark lateral line neuromasts. On the skin surface potential parallel DA innervation of trigeminal projections (TP) are labeled. Parallel innervation of central lateral line projection in the rhombencephalon is also labeled (CRhP). In the hindbrain most catecholaminergic cell bodies show TH-immunoreactivity but no GFP expression. **(D)** Z-MIP of otic slice stack “D” indicated in **(B)** (slice 125–185), showing ac and pc, also posterior to otic capsule the posterior lateral line ganglion (PG) and posterior lateral line nerve (PLLN) with parallel DA innervation (arrowheads). Asterisk marks one of the medial neuromasts on the surface of the OC. **(E)** Otic slice stack “E” indicated in **(B)** (slice 185–245) with innervation of sensory epithelia (arrowheads). **(F)** Otic slice stack “F” as indicated in **(B)** (slice 245–300) with sensory epithelia of maculae and innervation (arrowheads). In **(A–F)** asterisks mark neuromasts of the lateral line. Scale bars **(A)** 100 μm, **(B–F)** 50 μm. eGFP-CAAX-/SypGFP-expression driven by th:Gal4-VP16: green and panels 2; anti-TH: magenta and panels 3; TOTO-3: blue and panels 4.

We next looked at peripheral CA fibers, which extend to parts of the trigeminal system and inner ear sensory epithelia (Figure 8). We observe very fine arborizing CA fibers that cover larger areas of larval surface tissue, and potentially run in parallel with free trigeminal nerve endings in the skin (Figure 8A; Supplemental Movie 1 at levels 20 and 39). Such free nerve endings originate from sensory neurons located in the trigeminal ganglion (Pan et al., 2012). A confocal z-stack of the dorsal head region reveals *th:Gal4* driven GFP labeling in thin fibers in the skin above the rhombencephalon (Figure 8A2), which overlaps with faint TH-immunoreactivity (Figure 8A3).

We also looked for potential CA innervation of the inner ear sensory epithelia. The TOTO-3 nuclear label reveals the structure of the developing membranous labyrinth in the otic capsule (Figure 8B). A dorsal view of the lateral head shows the location of the inner ear, CA fibers projecting to neuromasts of the LL, and arborizing innervation in the lateral rhombencephalon (Figure 8C). Further ventral we observe the sectioned anterior and posterior canal, and CA fibers projecting to the posterior lateral line (PLL) ganglion and running in parallel with the PLL nerve, as well as fibers in the rhombencephalon in the region of the MON (Figure 8D). In the medial region of the developing membranous labyrinth we see the sectioned anterior, posterior and lateral canals and sensory epithelia of the utricular macula, as well as lateral, anterior and posterior cristae receiving innervation by CA fibers, as revealed by *th:Gal4* driven GFP expression (Figure 8E). In the ventral part of the membranous labyrinth, we additionally observe CA fibers innervating the saccular macula (Figure 8F).

### Catecholaminergic projections to the intestine

**Figure 9:**
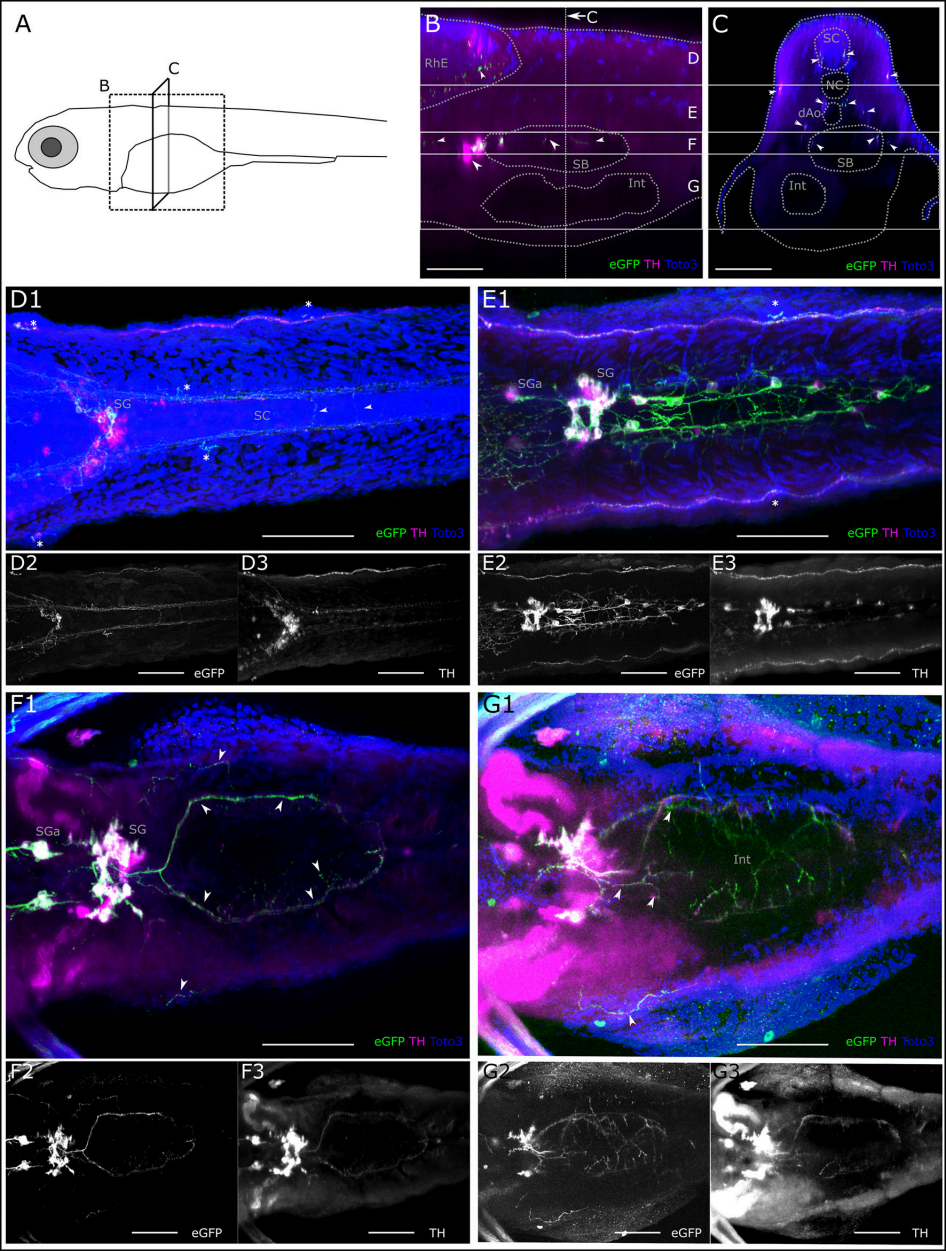
Catecholaminergic innervation of the intestine. **(A)** Schematic of 6dpf zebrafish larva with scanned sections as depicted in **(B,C)**. **(B)** Sagittal section from XZ-orthoslice at 243 μm of z-stack (dorsal scan, step size: 1 μm, total depth: 507 μm, total width: 509 μm) of larval region including the gut. Dotted gray lines outline anatomical landmark structures rhombencephalon (RhE), swim bladder (SB) and intestine (Int). Dashed white vertical line indicates region of transverse section shown in **C**. Solid white horizontal lines indicate borders of regions used for Z-MIPs shown in **(D–G)**. White arrowheads point to catecholaminergic structures in the RhE (region **D**), and in and around the SB, cell bodies anteriorly of the SB represent sympathetic ganglia (SG) and their projections (region **F**). **(C)** Transverse section from YZ-orthoslice at 246 μm of same z-stack. Outlined structures: spinal cord (SC), notochord (NC), dorsal Aorta (dAo), SB and Int. Arrowheads point to catecholaminergic projection tracts in SC (region **D**), and projections on lateral line nerve (regions **(D,E)**), around dAo (region **E**), and SB (region **F**). **(D)** Z-MIP from dorsal subregion as indicated in **(B,C)** (slice 1–140). Arrowheads point to catecholaminergic commissures of descending central projections in the SC. Asterisks mark neuromasts of the lateral line. Cell bodies are visible in the sympathetic ganglia (SG), of which only a subset is marked with GFP. **(E)** Z-MIP from middle substack (slice 140–225) containing catecholaminergic cell bodies in anterior SG (SGa) and in the more posteriorly located SG of the superior cervical ganglion complex, as well as dispersed caudal trunk sympathetic neurons, which project between NC and dAo. **(F)** Z-MIP of ventral substack (225–265) including cell bodies of SGa and SG, arrowheads point to ring-shaped innervation around SB and projections peripheral to SB. **(G)** Z-MIP of ventral-most substack (225–400) containing a ventral part of the gut. Arrowheads point to sparse catecholaminergic projections above and around the intestine. All scale bars **(B–G)**: 50 μm; GFP-expression driven by th:Gal4: green and panels 2; anti-TH: magenta and panels 3; TOTO-3: blue.

Since sympathetic catecholaminergic projections have not been analyzed in detail in zebrafish so far, we also briefly report our observation from the *Tg(th:Gal4-VP16)*^*m*1233^ and *Tg(UAS:eGFP-CAAX)*^*m*1230^ larva. Deep in the trunk, we observe rostral and caudal projections originating from sympathetic CA neurons located in the cervical region. Using the CLARITY method, we were able to observe these CA projections in the intestinal region (Figure 9 and Supplemental Figure S2). A sagittal orthoslice made from a confocal z-stack reveals both central and peripheral CA projections. In dorsal areas, we confirm the descending A11-type neuron projections into the rhombencephalon and spinal cord. In the center of the trunk, the somata of sympathetic neurons in the superior cervical ganglion complex project toward the swim bladder (Figure 9B). A transverse orthoslice made from the z-stack allowed us to observe fibers between the notochord and dorsal aorta, as well as fibers lateral of the swim bladder, which originate from the cervical sympathetic neurons (An et al., 2002). We also observe central descending fibers in the spinal cord, and peripheral fibers along the LL nerve originating posterior tubercular DA neurons (Figure 9C). In dorsal view z-projections of dorsal substack levels, we observe central descending fibers of PT neurons to form commissures in the spinal cord (Figure 9D). In more ventral substack z-projections between notochord and dorsal aorta, we observe fibers of sympathetic cervical neurons (Figure 9E). Further rostral, anterior of the cervical ganglion, we see an additional pair of anterior sympathetic ganglia. Caudal of the cervical ganglion, caudal trunk sympathetic neurons are located (Figure 9E). Caudal trunk sympathetic neurons, neurons of the cervical ganglion, or both, project in a ring-like fashion around the swim bladder, and fine fibers can be observed peripheral to the swim bladder (Figure 9F). Ventral of the swim bladder we observe further faint GFP and TH positive CA fibers (Figure 9G). CA fibers densely innervating a globular structure, probably representing the pancreas, for which CA innervation has been suggested in mammals (Zern et al., 1979; Fujimoto et al., 1993), also extends from the SG (Supplemental Figure 2).

## Discussion

In this study, we provide a comprehensive description of the peripheral projections of the diencephalic dopaminergic neurons with origin in the PT, which descend into the spinal cord and are homologous to the mammalian A11 group. In mammals, the A11 dopaminergic group, with somata located in the thalamus, provides the only source of DA in the spinal cord and has ascending projections into the telencephalon (Björklund and Skagerberg, 1979; Takada et al., 1988; Takada, 1993). We employed transgenic GFP expression in tyrosine hydroxylase positive neurons and immunohistochemical methods to detect tyrosine hydroxylase in catecholaminergic neurons, and the Islet1/Islet2 proteins in sensory afferents as well as antero- and retrograde labeling of sensory afferent and efferent neurons. We combined our immunohistochemical approach with the recently developed tissue clearing method CLARITY (Chung and Deisseroth, 2013; Tomer et al., 2014; Treweek et al., 2015) adapted for larval zebrafish. We were able to show that the posterior tubercular DA neurons of the rostral and caudal cluster (PTar and PTac, respectively) project to all neuromasts of the lateral line, the sensory epithelia of the inner ear, as well as to the lateral line ganglia and possibly other cranial ganglia. We have further documented arborizing DA fibers overlapping with the central projection field of the lateral line afferent neurons in the MON in the rhombencephalon. In addition, we have provided a description of catecholaminergic innervation of the developing intestine and swim bladder.

### Lateral line organ innervation by dopaminergic efferent neurons

We show that PTar and PTac DA neurons innervate all neuromasts of the anterior and PLL. Thus, the different anatomical subgroups of the A11-type DA system in zebrafish appear to differentially contribute to lateral line system innervation: for the posterior anatomical subgroups PTp (DC5) and PTN (DC6), we did not detect fibers projecting to the lateral line. For the PLL system, it appears that on each side of the larva at least three PTar and/or PTac DA neurons send efferent projections into the lateral line nerve, and that distinct subsets of lateral line organs may be innervated by individual DA neurons. However, our analysis did not detect any stereotypical pattern that would determine the lateral neuromast targeting of DA axons, since the variegated expression limited our analysis, and we could not obtain sufficient numbers to exclude any selective targeting. Synaptophysin-GFP expression in DA neurons revealed that the DA projections likely establish pre-synaptic structures at the base of cells in the center of the neuromast, presumably the hair cells. Since our GFP markers have variegated expression due to the Gal4:UAS system used, we cannot determine if each hair cell in a neuromast received DA innervation. A recent study labeled ribbon-synapses in the hair cells and showed that synaptophysin-positive terminals lie in proximity to the Ribeye signal, but did not find a direct overlap (Toro et al., 2015). The authors conclude that DA might be released in paracrine fashion around the hair cells. Experiments labeling post-synapse specific proteins in the hair cells could provide insight into whether synapses exist. Paracrine DA release has also been suggested in the saccule of the midshipman, where TH-positive neurons form swellings in proximity to the hair cells but no direct synapse can be observed (Perelmuter and Forlano, 2017). Efferent innervation of the lateral line neuromasts originating from cells located in the diencephalon was described decades ago in zebrafish larvae (Metcalfe et al., 1985) and other fish species (Zottoli and Van Horne, 1983; Puzdrowski, 1989; New and Singh, 1994). Dye-injections into the lateral line nerve of larval zebrafish labeled a few neurons in the diencephalon displaying TH-immunoreactivity, leading to the conclusion that these neurons are dopaminergic (Bricaud et al., 2001). However, these authors had not assigned the dopaminergic efferent lateral line neurons to any specific anatomical group. By labeling of afferent and efferent neurons, which contact a neuromast in the PLL, we were able to identify the efferent neurons in the diencephalon as neurons of the PTar and PTac cluster. Two recent studies, which focused primarily on physiological function, have also identified PTar and PTac (DC2 and DC4) as the ones contacting the lateral line neuromasts and the inner ear sensory epithelia (Jay et al., 2015; Toro et al., 2015). The neurons of these clusters were further found to respond to mechanosensory stimuli and be tuned to lateral line mediated stimulation (Reinig et al., 2017). On the functional aspect, one study reported an excitatory effect of dopamine release on hair cell activity mediated via D1 receptors (Toro et al., 2015). Other studies that measured activity in DA neurons found them to respond during fictive swimming (Jay et al., 2015), and to be inversely tuned to the velocity of a tactile stimulus exciting the lateral line (Reinig et al., 2017). The latter findings could be interpreted to point to an inhibitory modulation preventing self-stimulation of the sensory neurons during swimming (Feitl et al., 2010), but possibly also reflect a more complex gain control mechanism.

### Dopaminergic innervation of the inner ear

We observe catecholaminergic fibers projecting to the inner ear sensory epithelia, which most likely stem from the posterior tubercular DA neurons. In other teleosts, like the midshipman, catecholaminergic projections to the inner ear and to central neurons of the auditory and vocal circuitry have been described (Forlano et al., 2014; Perelmuter and Forlano, 2017). A DA efferent system also exists in the mammalian cochlea (Eybalin et al., 1993). In the guinea pig cochlea, DA depresses the spike rate in afferent fibers via D1 and D2 receptors (Oestreicher et al., 1997), which may provide a tonic inhibition to the auditory nerve to prevent excitotoxicity (Ruel et al., 2001). In mice, TH positive terminals are found intermingled with cholinergic terminals onto inner hair cells as part of the olivocochlear efferent bundle (Darrow et al., 2006; Maison et al., 2012; Nevue et al., 2016). Similarly, in the zebrafish lateral line the DA efferent system appears to exist alongside a cholinergic system (Flock and Lam, 1974; Bricaud et al., 2001). Ventral projections to the inner ear epithelia in the zebrafish may originate from the superior cervical ganglion besides projections from the PT (compare Figure 8F), and be therefore noradrenergic; however, this would have to be investigated in detail in a future study. In the trout saccule hair cells have dopamine receptors, and evidence for an adenylyl cyclase pathway with adenylyl cyclase isoforms and G protein alpha units that act in dopamine receptor signal transduction was found (Drescher et al., 2010).

### Dopaminergic innervation of the trigeminal system and sensory afferents

We found catecholaminergic fibers that appear to run in parallel with the patterns that have been described for free nerve endings of the trigeminal system (Sagasti et al., 2005) and to the trigeminal ganglion. We suggest that these projections originate from the posterior tubercular far projecting DA neurons, since they extend from the axonal projection tracts innervating the neuromasts. In the mammalian system, besides a direct innervation of sensory cells, there is evidence for DA modulation of sensory afferents in rats, where primary afferents in the mesencephalic trigeminal nucleus receive input from a DA fiber plexus extending over the locus coeruleus and nucleus parabrachialis (Copray et al., 1990). Besides the DA fibers projecting to the trigeminal ganglion, we observe that the fibers that innervate the neuromasts also innervate the lateral line ganglia extensively, where they run in between the cell bodies and exhibit structures that could be interpreted as growth cones or forming synapses. We also observe dense DA arborizations in parallel with the projection fields of the lateral line and inner ear sensory afferent neurons in the region of the MON in the rhombencephalon. This emphasizes that DA modulation acts at different levels of the sensory circuit. In experiments labeling all cranial ganglia with the islet1/2 antibody, we further observed projections along the vagal ganglia and probably the otic ganglion. DA modulation by A11 neurons has been demonstrated to be involved in pain modulation in mammals, where they inhibit the trigeminal cervical complex in response to noxious stimuli of the ophthalmic dermatome, mediated by D2 receptors (Charbit et al., 2009). Further, sensory perception of different modalities is affected in Parkinson's patients (Conte et al., 2013).

### Dopaminergic innervation of the abdomen

Taking advantage of the CLARITY technique, we were able to describe catecholaminergic projections to the larval inner organs. So far, there is no detailed description of catecholaminergic innervation of the larval zebrafish abdomen. Here, we also did not focus on the innervation of the inner organs, but briefly describe putative noradrenergic sympathetic innervation of the intestine, swim bladder, and pancreas by neurons with cell bodies in the superior cervical ganglion complex. Catecholaminergic innervation of the pancreas has been previously described in mammals (Zern et al., 1979; Fujimoto et al., 1993). We also observe catecholaminergic projections along the dorsal aorta. We further see faint catecholaminergic innervation in the periphery of the intestine.

### Implications for dopaminergic signaling during development

While the descending A11 system in mammals projects into the spinal cord, the A11-type posterior tubercular projections in teleosts appear to extensively innervate peripheral sensory organs as others and we have shown (Jay et al., 2015; Toro et al., 2015). However, a number of studies trying to elucidate the physiological function of dopamine signaling in the zebrafish larva have focused only on the relevance for the developing motor circuits (Thirumalai and Cline, 2008; Lambert et al., 2012). With increasing evidence for an important role of dopamine modulation of sensory systems, both directly (e.g., in fish Toro et al., 2015; Forlano and Sisneros, 2016; and indirectly, e.g., in mice Gittelman et al., 2013; Nevue et al., 2015, 2016), we should consider the impact of dopamine on those systems. This means, in behavioral studies results have to be interpreted in a way where sensing as well as motor behavior are potentially affected by DA modulation. In some cases, this may make the interpretation of results more difficult. The emerging picture of the DA system in the zebrafish emphasizes the need for studies that can consider the physiological effects of dopamine on motor- as well as sensory circuits.

## Author contributions

MH-T, MB, IS, and LT: Performed all experiments and recorded data; AF: Generated crucial transgenic zebrafish lines; MH-T, IS, and LT: Analyzed data; MH-T: Assembled all figures; MH-T and WD: Wrote the manuscript; WD: Designed the study.

### Conflict of interest statement

The authors declare that the research was conducted in the absence of any commercial or financial relationships that could be construed as a potential conflict of interest.

## Abbreviations

AC: amacrine cells
ac: anterior canal
acr: anterior crista
AG: anterior lateral line ganglion
AGa: anterior part of AG
AGm: medial part of AG
ALL: anterior lateral line
CB: carotid body
CRhP: central rhombencephalic projection
D: dorsal line of NM
dAo: dorsal aorta
DRG: dorsal root ganglia
VII. G: facial ganglion
VIII.G: statoacoustic ganglion
IX.G: glossopharyngeal ganglion
X.G: vagus ganglion
H: hypothalamus
Hc: DA neurons of caudal Hypothalamus
Hdm: DA neurons of dorsal medial Hypothalamus
IO: infraorbital line of NM
Int: intestine
lc: lateral canal
lcr: lateral crista
LL: lateral line
L: lateral line of lateral NM
LII: secondary lateral line of NM
M: mandibular line of NM
MC: Mauthner cell
MI: middle line of NM
MON: medial octavolateralis nucleus
N: nasal line of NM
NC: notochord
NM: neuromasts
O: otic line of NM
OB: olfactory bulb
OC: otic capsule
OE: otic epithelium
OP: opercular line of NM
Pa: Pancreas
pc: posterior canal
pcr: posterior crista
PF: pectoral fin
PG: posterior lateral line ganglion
PGm: medial PG
PLL: posterior lateral line
PLLN: posterior lateral line nerve
PO: preoptic region
PR: pretectum
PTac: caudal subcluster of anterior PT DA cluster
PTar: rostral subcluster of anterior PT DA cluster
PTp: posterior PT DA cluster
PTN: posterior tuberal nucleus DA cluster
PT: posterior tuberculum
REN: rhombencephalic efferent neurons
RhE: rhombencephalon
SB: swim bladder
SC: spinal cord
SG: sympathetic ganglia
sm: saccular macula
SO: supraorbital line of NM
SP: subpallium
Tec: tectum
TG: trigeminal ganglion
TP: trigeminal projections
um: utricular macula
CA: catecholaminergic
DA: dopaminergic
DASPEI: 2-(4-(dimethylamino)styryl) -N-Ethylpyridinium Iodide
dpf: days post fertilization
eGFP-CAAX: membrane-tagged enhanced Green Fluorescent Protein
GFP: green fluorescent protein
HM: hydrogel monomer
ISL: Islet1 or Islet2 transcription factor
ir: immuno-reactive
MIP: maximum intensity projection
NA: noradrenergic
Otp: Orthopedia transcription factor
PTU: phenylthiourea
RFP: red fluorescent protein
RH: Rhodamine Dextran
SypGFP: synaptophysin eGFP fusion protein
TH: tyrosine hydroxylase.
