# Supplementary material for: Projections of the Diencephalospinal Dopaminergic System to Peripheral Sense Organs in Larval Zebrafish (Danio rerio)
Source: Front Neuroanat. 2018 Mar 19;12:20. doi: 10.3389/fnana.2018.00020 (PMC5868122; doi:10.3389/fnana.2018.00020)
Supplement: Supplementary file 1 [file Presentation1.PDF]

Supplementary Material

Projections of the diencephalospinal dopaminergic system to peripheral sense organs in larval zebrafish (*Danio rerio*)

Melanie Haehnel-Taguchi*, António M. Fernandes, Margit Böhler, Ina Schmitt, Lena Tittel, and Wolfgang Driever*

*** Correspondence:** melanie.haehnel@biologie.uni-freiburg.de or driever@biologie.uni-freiburg.de

1 Inventory of Supplementary Data

Supplemental Figure 1: Dopaminergic innervation of lateral line ganglia

Supplemental Figure 2: Catecholaminergic innervation of the abdomen, ventral scan.

Supplemental Table 1: Recording conditions of confocal stacks used in this study

Supplemental Movie 1: 6 dpf whole mount larva with catecholaminergic system labeled

2 Supplementary Figures and Tables

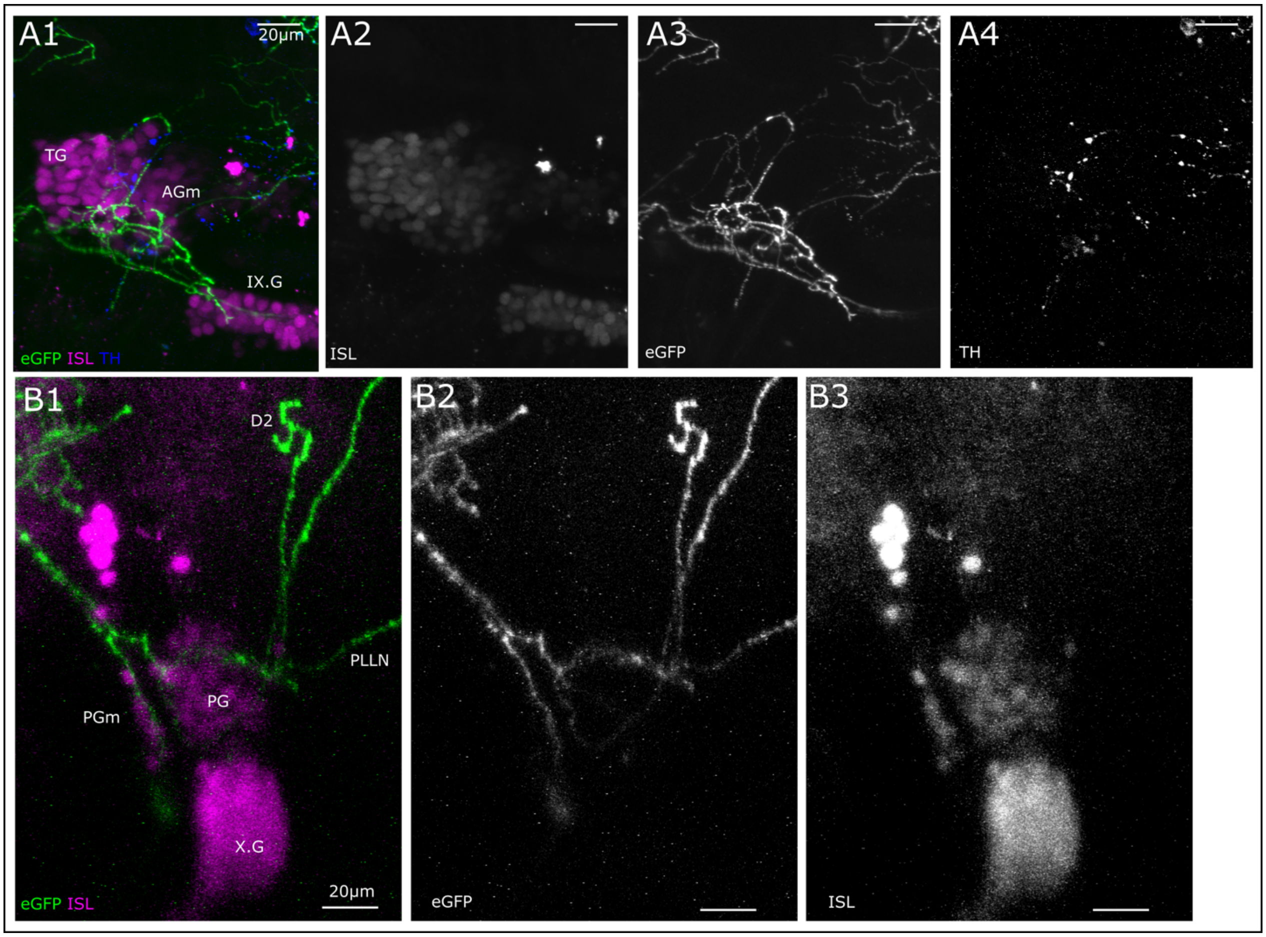

Supplemental Figure 1

Dopaminergic innervation of lateral line ganglia.

A. Lateral view, MIP (total depth of 54.19 µm, step size: 1.18 µm), of region of medial anterior lateral line ganglion (mAG) with trigeminal ganglion (TG) and glossopharyngeal ganglion (IX.G). GFP expression driven by *th:Gal4-VP16* (green, A3) in projections targeting the trigeminal and lateral line ganglion and also running through IX.G. Cell bodies of sensory afferent neurons in ganglia marked with Islet1/Islet2 immunoreactivity (ISL, magenta, A2). TH-immunoreactivity shown in blue and A4. **B.** Lateral view, MIP (total depth of 40.95 µm, step size 1.17 µm), of region of posterior lateral line ganglion, which can be further subdivided into medial posterior lateral line ganglion (PGm) and posterior lateral line ganglion (PG). Also visible: caudal vagus ganglion (X.G). Neurons with GFP expression (green and B2) target PGm and PG and project to dorsal neuromast, along central projection of lateral line afferent neurons and posterior lateral line nerve (PLLN). Sensory afferent neurons marked with ISL-immunoreactivity (magenta and B3). All scale bars: 20µm.

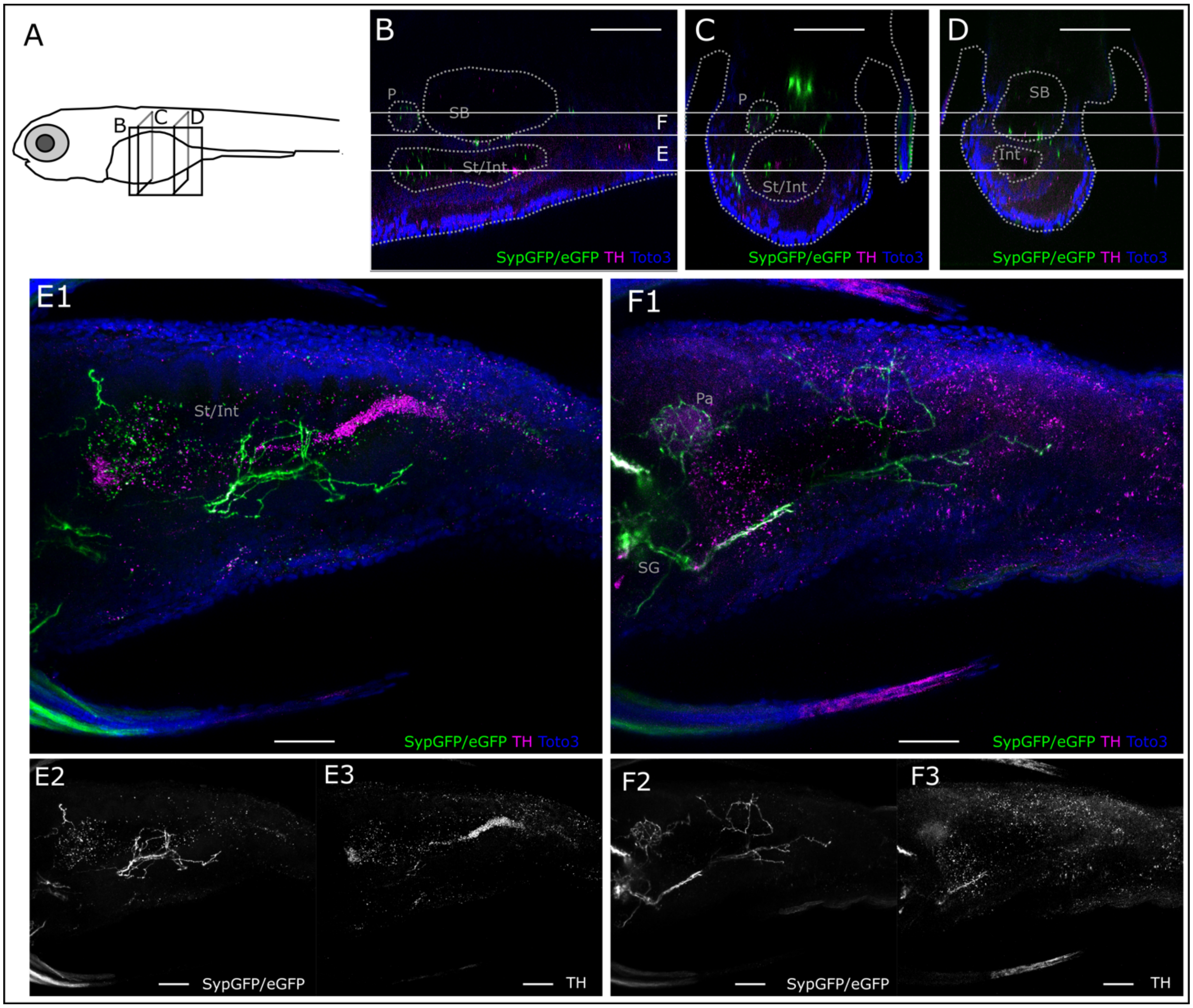

Supplemental Figure 2

Catecholaminergic innervation of the abdomen, ventral scan.

A. Schematic of 6dpf zebrafish larva with scanned sections as depicted in B-D. **B.** Sagittal section from XZ-orthoslice at 272 µm of z-stack (ventral scan, step size: 1µm, total depth: 390 µm, total width: 509 µm) of larval region including the gut. Dotted grey lines outline anatomical landmark structures, probably the pancreas (P) swim bladder (SB) and stomach and intestine (St/Int). Solid white horizontal lines indicate borders of regions used for MIPs shown in E and F. **C.** Transverse section from YZ-orthoslice at 100µm of same z-stack. Outlined structures: P and St/Int. **D.** Transverse section from YZ-orthoslice at 381µm of same z-stack. Outlined structures: SB and Int. **E.** MIP from subregion as indicated in B-D (slices 170-230). Catecholaminergic projections between and around the SB and the Int/St are labeled with GFP driven by th:Gal4-VP16 and are faintly TH-immuno-reactive. **F.** MIP from subregion as indicated in B-D (slices 230-270). Catecholaminergic projection around the SB is labeled with GFP and anti-TH. Round structure rostral to SB shows strong GFP and anti-TH labeled innervation, probably representing innervation of the pancreas (P) by sympathetic projections. All scale bars: 50µm. GFP: green, panels 2, anti-TH: magenta, panels 3, TOTO-3: blue.

Supplemental Table 1

Recording conditions of confocal stacks used in this study

The table is provided on the next page of this PDF.

Supplemental Movie 1

6 dpf whole mount larva with catecholaminergic system labeled

Image stack of larval whole mount at 6dpf of transgenic line (505 sections, stepsize 1µm). GFP expression driven by *th:Gal4-VP16* shown in green, TH-immunoreactivity shown in magenta. Dorsal section (1-30) show labeled innervation of the skin, possibly representing parallel innervation of free nerve endings. Starting at section 30 catecholaminergic cell bodies in the medulla oblongata (MO) become visible. Approximately, between sections 40-120 an ectopically labeled commissure can be observed. Section 112-167 reveals the double labeled posterior lateral line nerve (PLLN). Sections 158-198 show TH-immunoreactive cells in the locus coeruleus (LC) of which a few are also GFP positive. In sections, 173-272 cell bodies and projections of the sympathetic ganglia (SG) can be observed. Sections 232-300 reveal the diencephalic dopaminergic clusters (PTar, PTac, PTp, PTN, Hdm and Hc). In sections, 425-503 cells of the carotid body (CB) are labeled.

Supplemental Table 1													
Figure	Date	Lines			Antibodies/ cell label			Orientiation	Microscope	Resolution		Tile of Tiles	Zoom	Notes
1A	11.03.16	m1233	m1230	m1238	GFP, Alexa 488	TH, Alexa 555	Toto3 (633)	dorsal	LSM-510 upright	1024x1024 px	1 px = 0,497 µm	1, 2, 3 of 3	0,7	Z-stack, 410µm, Stepsize 1µm
1B	01.03.16	m1233	m1230	m1238	GFP, Alexa 488	TH, Alexa 555	Toto3 (633)	ventral	LSM-510 upright	1024x1024 px	1 px = 0,497 µm	1 ,2 ,3 of 4	0,7	Z-Stack, 390µm, Stepzise 1µm
1C	03.09.15	m1233	m1230		GFP, Alexa 488	TH, Alexa 555	Toto3 (633, not shown)	dorsal	LSM-510 upright	512x512 px	1 px =0,994 µm	1, 2 of 3	0,7	Z-Stack, 180 to 240 of total 507µm, stepsize: 1µm
2A (1-4')	11.05.16	m1233	m1230	m1238	GFP, Alexa 488	TH, Alexa 633	Rhod. Dextran (555)	dorsal	LSM-510 upright	1024x1024 px	1px = 0,465 µm	1 of 2	0,7	Z-Stack: 55µm, step size: 1µm
2A (1'-4')	11.05.16	m1233	m1230	m1238	GFP, Alexa 488	TH, Alexa 633	Rhod. Dextran (555)	dorsal	LSM-510 upright	1024x1024 px	1px = 0,465 µm	2 of 2	0,7	Z-Stack: 70µm, step size: 1µm
2B (1-4)	11.05.16	m1233	m1230	m1238	GFP, Alexa 488	TH, Alexa 633	Rhod. Dextran (555)	dorsal	LSM-510 upright	1024x1024 px	1px = 0,465 µm	1 of 2	0,7	Z-Stack: 16µm, step size: 1µm
2C (1-4)	11.05.16	m1233	m1230	m1238	GFP, Alexa 488	TH, Alexa 633	Rhod. Dextran (555)	dorsal	LSM-510 upright	1024x1024 px	1px = 0,465 µm	1 of 2	0,7	Z-Stack: 16µm, step size: 1µm
2D (1-4)	12.05.16	m1233	m1230	m1238	GFP, Alexa 488	TH, Alexa 633	Rhod. Dextran (555)	dorsal	LSM-510 upright	1024x1024 px	1px = 0,497 µm	1 of 1	0,7	Z-Stack: 35µm, step size: 1µm
2E (1-4)	12.05.16	m1233	m1230	m1238	GFP, Alexa 488	TH, Alexa 633	Rhod. Dextran (555)	dorsal	LSM-510 upright	1024x1024 px	1px = 0,497 µm	1 of 1	0,7	Z-Stack: 17µm, step size: 1µm
2F (1-4)	18.05.16	m1233	m1230	m1238	GFP, Alexa 488	TH, Alexa 633	Rhod. Dextran (555)	dorsal	LSM-510 upright	1024x1024 px	1px = 0,497 µm	1 of 1	0,7	Z-Stack: 35µm, step size: 1µm
2F (1-4)	18.05.16	m1233	m1230	m1238	GFP, Alexa 488	TH, Alexa 633	Rhod. Dextran (555)	dorsal	LSM-510 upright	1024x1024 px	1px = 0,497 µm	1 of 1	0,7	Z-Stack: 13µm, step size: 1µm
3B2	11.03.16	m1233	m1230	m1238	GFP, Alexa 488	TH, Alexa 555	Toto3 (633)	dorsal	LSM-510 upright	1024x1024 px	1 px = 0,497 µm	1, 2 of 3	0,7	Z-Stack: 477µm, step size: 1µm
3C2	01.03.16	m1233	m1230	m1238	GFP, Alexa 488	TH, Alexa 555	Toto3 (633)	ventral	LSM-510 upright	1024x1024 px	1 px = 0,497 µm	1, 2 of 4	0,7	Z-Stack: 434µm, step size: 1µm
4A (1-5)	11.03.16	m1233	m1230	m1238	GFP, Alexa 488	TH, Alexa 555	Toto3 (633)	dorsal	LSM-510 upright	12024x1224 px	1 px = 0,497 µm	1, 2 of 4	0,7	Z-Stack: 135µm, step size: 1µm
4B (1-5)	11.03.16	m1233	m1230	m1238	GFP, Alexa 488	TH, Alexa 555	Toto3 (633)	dorsal	LSM-510 upright	12024x1224 px	1 px = 0,497 µm	2 of 4	0,7	Z-Stack: 122µm, step size: 1µm
4C (1-5)	11.03.16	m1233	m1230	m1238	GFP, Alexa 488	TH, Alexa 555	Toto3 (633)	dorsal	LSM-510 upright	12024x1224 px	1 px = 0,497 µm	1 of 4	0,7	Z-Stack: 122µm, step size: 1µm
4D (1-5)	01.03.16	m1233	m1230	m1238	GFP, Alexa 488	TH, Alexa 555	Toto3 (633)	ventral	LSM-510 upright	1024x1024 px	1 px = 0,497 µm	1, 2, 3 of 4	0,7	Z-Stack: 91µm, step size: 1µm
4E (1-5)	01.03.16	m1233	m1230	m1238	GFP, Alexa 488	TH, Alexa 555	Toto3 (633)	ventral	LSM-510 upright	1024x1024 px	1 px = 0,497 µm	1, 2, 3 of 4	0,7	Z-Stack: 226µm, step size: 1µm
5A (1-5)	11.03.16	m1233	m1230	m1238	GFP, Alexa 488	TH, Alexa 555	Toto3 (633)	dorsal	LSM-510 upright	12024x1224 px	1 px = 0,497 µm	3, 4 of 4	0,7	Z-Stack: 175µm, step size: 1µm
5B (1-5)	11.03.16	m1233	m1230	m1238	GFP, Alexa 488	TH, Alexa 555	Toto3 (633)	dorsal	LSM-510 upright	12024x1224 px	1 px = 0,497 µm	4 of 4	0,7	Z-Stack: 130µm, step size: 1µm
5C (1-5)	11.03.16	m1233	m1230	m1238	GFP, Alexa 488	TH, Alexa 555	Toto3 (633)	dorsal	LSM-510 upright	12024x1224 px	1 px = 0,497 µm	4 of 4	0,7	Z-Stack: 60µm, step size: 1µm
5D (1-5)	23.03.16	m1233	m1230	m1238	GFP, Alexa 488	TH, Alexa 555	Toto3 (633)	lateral	LSM-510 upright	1024x1024 px	1 px = 0,329 µm	1 of 1	1	Z-Stack: 25µm, step size: 1µm
6A (1-4)	24.03.16		m1230	m1238	GFP, Alexa 488	TH, Alexa 555	Toto3 (633)	lateral	LSM-510 upright	1024x1024 px	1px = 0,164 µm	1 of 1	2	Z-Stack: 47µm, step size: 1µm
6B (1-4)	24.03.16		m1230	m1238	GFP, Alexa 488	TH, Alexa 555	Toto3 (633)	dorsal	LSM-510 upright	1024x1024 px	1 px = 0,168 µm	1 of 1	2	Z-Stack: 59µm, step size: 1µm
6C (1-4)	30.05.16	m1233	m1230	m1238	GFP, Alexa 488	TH, Alexa 633	Rhod. Dextran (555)	lateral	LSM-510 upright	1024x1024 px	1px = 0,088 µm	1 of 1	4	Z-stack: 34µm, step size: 1µm
6D (1-4)	30.05.16	m1233	m1230	m1238	GFP, Alexa 488	TH, Alexa 633	Rhod. Dextran (555)	lateral	LSM-510 upright	1024x1024 px	1px = 0,088 µm	1 of 1	4	Z-stack: 37µm, step size: 1µm
7A (1-4)	30.05.16	m1233	m1230	m1238	GFP, Alexa 488	TH, Alexa 633	Rhod. Dextran (555)	lateral	LSM-510 upright	1024x1024 px	1px = 0,497 µm	1 of 1	0,7	Z-stack: 81µm, step size: 1µm
7B (1-4)	30.05.16	m1233	m1230	m1238	GFP, Alexa 488	TH, Alexa 633	Rhod. Dextran (555)	lateral	LSM-510 upright	1024x1024 px	1px = 0,497 µm	1 of 1	0,7	Z-stack: 81µm, step size: 1µm
7C (1-4)	18.05.16	m1233	m1230	m1238	GFP, Alexa 488	TH, Alexa 633	Rhod. Dextran (555)	dorsal	LSM-510 upright	1024x1024 px	1px = 0,176 µm	1 of 1	2	Z-Stack: 59µm, step size: 1µm
7D (1-4)	14.06.16	m1233	m1230	m1238	GFP, Alexa 488	TH, Alexa 633	Rhod. Dextran (555)	dorsal	LSM-510 upright	1024x1024 px	1px = 0,497 µm	1 of 1	0,7	Z-Stack: 155µm, step size: 1µm
7E (1-3)	02.08.16	m1233	m1230		GFP, Alexa 488	TH, Alexa 633 (n.S)	Islet AB, Alexa 555	dorsal	LSM-510 upright	1024x1024 px	1px = 0,465 µm	1, 2 of 2	0,7	Z-Stack: 476µm, step size 1µm
7F (1-4)	03.08.16	m1233	m1230		GFP, Alexa 488	TH, Alexa 633	Islet AB, Alexa 555	dorsal	LSM-510 upright	1024x1024 px	1px = 0,465 µm	1 of 1	0,7	Z-Stack: 137.9µm, step size: 3.63 µm,
8A (1-3)	03.09.15	m1233	m1230		GFP, Alexa 488	TH, Alexa 555	Toto3 (633)	dorsal	LSM-510 upright	512x512 px	1px = 0,994 µm	1, 2 of 3	0,7	Z-Stack, 16-40,step size 1µm
8B	11.03.16	m1233	m1230	m1238	GFP, Alexa 488	TH, Alexa 555	Toto3 (633)	dorsal*	LSM-510 upright	1024x1024 px	1 px = 0,497 µm	2 of 3	0,7	Ortho Slice sagittal XZ 173, cropped
8C (1-4)	11.03.16	m1233	m1230	m1238	GFP, Alexa 488	TH, Alexa 555	Toto3 (633)	dorsal	LSM-510 upright	1024x1024 px	1 px = 0,497 µm	2 of 3	0,7	Z-Stack, 1-125, step size 1µm
8D (1-4)	11.03.16	m1233	m1230	m1238	GFP, Alexa 488	TH, Alexa 555	Toto3 (633)	dorsal	LSM-510 upright	1024x1024 px	1 px = 0,497 µm	2 of 3	0,7	Z-Stack, 125-185, step size 1µm
8E (1-4)	11.03.16	m1233	m1230	m1238	GFP, Alexa 488	TH, Alexa 555	Toto3 (633)	dorsal	LSM-510 upright	1024x1024 px	1 px = 0,497 µm	2 of 3	0,7	Z-Stack, 185-245, step size 1µm
8F (1-4)	11.03.16	m1233	m1230	m1238	GFP, Alexa 488	TH, Alexa 555	Toto3 (633)	dorsal	LSM-510 upright	1024x1024 px	1 px = 0,497 µm	2 of 3	0,7	Z-Stack, 245-300, step size 1µm
9B	03.09.15	m1233	m1230		GFP, Alexa 488	TH, Alexa 555	Toto3 (633)	dorsal*	LSM-510 upright	512x512 px	1px = 0,994 µm	3 of 3	0,7	Ortho Slice sagittal
9C	03.09.15	m1233	m1230		GFP, Alexa 488	TH, Alexa 555	Toto3 (633)	dorsal*	LSM-510 upright	512x512 px	1px = 0,994 µm	3 of 3	0,7	Ortho Slice transverse
9D (1-3)	03.09.15	m1233	m1230		GFP, Alexa 488	TH, Alexa 555	Toto3 (633)	dorsal	LSM-510 upright	512x512 px	1px = 0,994 µm	3 of 3	0,7	Z-Stack, 1-140, step size: 1µm
9E (1-3)	03.09.15	m1233	m1230		GFP, Alexa 488	TH, Alexa 555	Toto3 (633)	dorsal	LSM-510 upright	512x512 px	1px = 0,994 µm	3 of 3	0,7	Z-Stack, 140-225, step size: 1µm
9F (1-3)	03.09.15	m1233	m1230		GFP, Alexa 488	TH, Alexa 555	Toto3 (633)	dorsal	LSM-510 upright	512x512 px	1px = 0,994 µm	3 of 3	0,7	Z-Stack, 225-265, step size: 1µm
9G (1-3)	03.09.15	m1233	m1230		GFP, Alexa 488	TH, Alexa 555	Toto3 (633)	dorsal	LSM-510 upright	512x512 px	1px = 0,994 µm	3 of 3	0,7	Z-Stack, 265-400, step size: 1µm
S1A (1-4)	04.08.16	m1233	m1230		GFP, Alexa 488	TH, Alexa 633	Islet AB, Alexa 555	lateral	LSM-510 upright	1024x1024 px	1px = 0,164 µm	1 of 1	2	Z-Stack: 54,188µm, step size: 1,178µm
S1B (1-3)	04.08.16	m1233	m1230		GFP, Alexa 488	TH, Alexa 633 (n.S)	Islet AB, Alexa 555	lateral	LSM-510 upright	1024x1024 px	1px = 0,274µm	1 of 1	1,2	Z-Stack: 40,95µm, step size: 1,17µm
S2B	01.03.16	m1233	m1230	m1238	GFP, Alexa 488	TH, Alexa 555	Toto3 (633)	ventral*	LSM-510 upright	1024x1024 px	1 px = 0,497 µm	3 of 4	0,7	Ortho Slice sagittal
S2C	01.03.16	m1233	m1230	m1238	GFP, Alexa 488	TH, Alexa 555	Toto3 (633)	ventral*	LSM-510 upright	1024x1024 px	1 px = 0,497 µm	3 of 4	0,7	Ortho Slice tranverse rostral
S2D	01.03.16	m1233	m1230	m1238	GFP, Alexa 488	TH, Alexa 555	Toto3 (633)	ventral*	LSM-510 upright	1024x1024 px	1 px = 0,497 µm	3 of 4	0,7	Ortho Slice tranverse caudal
S2E (1-3)	01.03.16	m1233	m1230	m1238	GFP, Alexa 488	TH, Alexa 555	Toto3 (633)	ventral	LSM-510 upright	1024x1024 px	1 px = 0,497 µm	3 of 4	0,7	Z-Stack: 60µm, step size: 1µm
S2F (1-3)	01.03.16	m1233	m1230	m1238	GFP, Alexa 488	TH, Alexa 555	Toto3 (633)	ventral	LSM-510 upright	1024x1024 px	1 px = 0,497 µm	3 of 4	0,7	Z-Stack: 40µm, step size: 1µm
